# Differences in leaf thermoregulation and water use strategies between three co‐occurring Atlantic forest tree species

**DOI:** 10.1111/pce.13208

**Published:** 2018-05-03

**Authors:** Sophie Fauset, Helber C. Freitas, David R. Galbraith, Martin J.P. Sullivan, Marcos P.M. Aidar, Carlos A. Joly, Oliver L. Phillips, Simone A. Vieira, Manuel U. Gloor

**Affiliations:** ^1^ School of Geography University of Leeds Leeds LS2 9JT UK; ^2^ Departamento de Física, Faculdade de Ciências Universidade Estadual Paulista Av. Eng. Luiz Edmundo Carrijo Coube, 14‐01, Bauru São Paulo 17033‐360 Brazil; ^3^ Instituto de Botânica de São Paulo Avenida Miguel Stéfano São Paulo 04301‐902 Brazil; ^4^ Departamento de Biologia Vegetal, Instituto de Biologia Universidade Estadual de Campinas Rua Monteiro Lobato, Cidade Universitâria, Campinas São Paulo 13083‐862 Brazil; ^5^ Núcleo de Estudos e Pesquisas Ambientais Universidade Estadual de Campinas Rua dos Flamboyants, 155, Campinas São Paulo 13083‐867 Brazil

**Keywords:** boundary layer, energy balance, functional traits, leaf temperature, leaf width, montane, radiation, stomatal conductance, transpiration, tropical forest

## Abstract

Given anticipated climate changes, it is crucial to understand controls on leaf temperatures including variation between species in diverse ecosystems. In the first study of leaf energy balance in tropical montane forests, we observed current leaf temperature patterns on 3 tree species in the Atlantic forest, Brazil, over a 10‐day period and assessed whether and why patterns may vary among species. We found large leaf‐to‐air temperature differences (maximum 18.3 °C) and high leaf temperatures (over 35 °C) despite much lower air temperatures (maximum 22 °C). Leaf‐to‐air temperature differences were influenced strongly by radiation, whereas leaf temperatures were also influenced by air temperature. Leaf energy balance modelling informed by our measurements showed that observed differences in leaf temperature between 2 species were due to variation in leaf width and stomatal conductance. The results suggest a trade‐off between water use and leaf thermoregulation; Miconia cabussu has more conservative water use compared with Alchornea triplinervia due to lower transpiration under high vapour pressure deficit, with the consequence of higher leaf temperatures under thermal stress conditions. We highlight the importance of leaf functional traits for leaf thermoregulation and also note that the high radiation levels that occur in montane forests may exacerbate the threat from increasing air temperatures.

## Abstract 2

In the first study of leaf energy balance in tropical montane forests, we observed current leaf temperature patterns in the Atlantic forest, Brazil, and assessed whether and why patterns may vary among species. We found large leaf‐to‐air temperature differences that were influenced strongly by radiation and differences in leaf temperature between 2 species due to variation in leaf width and stomatal conductance. We highlight the importance of leaf functional traits for leaf thermoregulation and also note that the high radiation levels that occur in montane forests may exacerbate the threat from increasing air temperatures.

## 1 INTRODUCTION

The surface temperature of the Earth is increasing (Rahmstorf, Foster, & Cahill, 2017) and set to continue increasing into the future (Collins et al., 2013). The majority of tropical forests show a trend of increasing air temperature over the past 35 years, which is particularly strong in South America with recent increases up to 0.5 °C per decade (Figure S1). Temperature extremes are also increasing and are predicted to increase in the coming century (Coumou & Robinson, 2013). Although it is known that temperature influences plant functioning, the response of plants to increasing temperature and variation between species is a major uncertainty (Teskey et al., 2015). Tropical forests are particularly important in this regard as they are a considerable store of terrestrial carbon (Pan et al., 2011), potentially already function near their maximum temperature (Doughty & Goulden, 2008) and given their location cannot be replaced by species from lower latitudes. The biodiversity of tropical montane forests, which house many endemic species with restricted ranges, may be at particular risk from higher temperatures due to limits on upslope migration, especially for tree species in forests occurring on mountaintops (Phillips, 1997). Modelling studies suggest increasing temperatures are also likely to have a negative effect on tropical forest vegetation carbon; however, the extent of projected impact varies greatly between different models (Galbraith et al., 2010; Huntingford et al., 2013) as do the physiological mechanisms behind the declines (Galbraith et al., 2010).

Temperature can impact plant physiology both directly, by influencing rates of photosynthesis and respiration and indirectly by altering the ambient vapour pressure deficit (*D*; Lloyd & Farquhar, 2008). *D* increases with an increase in air temperature (*T*_A_) if relative humidity (*h*) stays constant, and stomatal conductance (*g*_s_) typically declines with increasing *D* (Leuning, 1995) to avoid excessive water loss. The reduction in *g*_s_ with *D* has the consequence of reduced CO_2_ concentration within the leaf. The photosynthetic optima of plants are typically near the mean maximum ambient temperature, showing the acclimation of plants to their environment (Slot & Winter, 2017). The temperature of the leaf tissue itself is the relevant temperature for the control of leaf physiological processes rather than *T*_A_. High leaf temperatures (*T*_L_) can induce damage to photosynthetic machinery; above *c*. 35 °C thylakoid membranes have been observed to structurally change (Gounaris, Brain, Quinn, & Willams, 1983, 1984), and above 40 °C photosystem II (PSII) may become deactivated and the electron transport rate reduced (Allakhverdiev et al., 2008). Chlorophyll fluorescence parameters to assess heat tolerance of PSII show critical temperature thresholds in the region of 45–60 °C, with significant variation between species (O'Sullivan et al., 2017; Sastry & Barua, 2017). Irreversible thermal damage to photosynthetic machinery has been observed to occur at 52 °C in a tropical species (Krause et al., 2010).

Extremes of microclimate can lead to leaf temperatures that are markedly different from *T*_A_. For example, leaf temperatures up to 10 °C above air temperatures when leaves were brightly lit have been observed in the Amazon (Doughty & Goulden, 2008) and in Panama (Rey‐Sanchez, Slot, Posada, & Kitajima, 2016), and Slot, Garcia, and Winter (2016) found leaf temperatures of a *Ficus insipida* regularly exceeded 40 °C and even reached 48 °C during a 3‐week period in Panama. Yet despite these striking patterns of leaf temperatures, and the on‐going and anticipated increases in air temperatures, there are few datasets examining fluctuations of leaf temperatures in situ in tropical forests and, to our knowledge, none in tropical montane forests.

Leaf energy balance theory can be used to address the drivers of *T*_L_ in a mechanistic approach (e.g., Michaletz et al., 2016). Developed from the Penman energy balance approach to evapotranspiration (Penman, 1948), the leaf energy balance equation (see Section 2, Equation (3)) estimates the leaf‐to‐air temperature difference (Δ*T*) for given microclimatic and leaf‐specific variables (Jones, 1992). The leaf energy balance shows that Δ*T* is dependent on the net energy provided (or lost) by radiation and the energy lost through transpiration. The effects of these fluxes on Δ*T* depend on leaf shape and physiology through the boundary layer and stomatal resistances to water transport. Stomatal resistance is dependent on stomatal activity and boundary layer resistance increases with leaf width (see Section 2). Hence, although Δ*T* is strongly influenced by microclimatic conditions (in particular radiation and *D*), leaf traits (width and stomatal conductance) can also play a role in regulation of leaf temperature. In addition, leaves can alter their physical position through changes in angle and/or orientation to increase or decrease the amount of radiation received.

Leaf structural traits (leaf mass per area [LMA] and leaf dry matter content [LDMC]) and stomatal conductance (*g*_s_) influence the time required for leaf temperature to change following a change in the environment (the thermal time constant [τ]; Jones, 1992). Leaves with a long τ will show smaller temperature changes in a fluctuating environment, maintaining the leaf temperature closer to the mean air temperature than a leaf with a small τ, which will track fluctuation in air temperature (Michaletz et al., 2015).

Given the diversity of leaf structures and physiology observed within and among tropical forest species (e.g., variation in leaf[let] area over five orders of magnitude for a large sample of tropical species; Wright et al., 2017), it is possible that there will be diversity in leaf strategies with regard to temperature (Michaletz et al., 2015). This means that the impacts of potential future environmental changes may vary between species even within a single biome. Future combined atmospheric changes of increasing CO_2_ and increasing *T*_A_ could be particularly important for *T*_L_, as plants tend to respond to increasing CO_2_ by reducing *g*_s_ (Way, Oren, & Kroner, 2015). Reducing *g*_s_ decreases water use but also has the consequence of increasing leaf temperature (Barker et al., 2005; Drake, Gonzàlez‐Meler, & Long, 1997) and can lead to premature leaf senescence under heat wave conditions (Warren, Norby, & Wullschleger, 2011). Increases in *T*_A_ could be particularly important under fluctuating and extreme conditions (e.g., heat waves), increasing the occurrence of leaves reaching or exceeding threshold temperatures resulting in leaf damage.

We present an observational study of leaf temperatures in a highly threatened tropical forest region—the Atlantic forest, among the most diverse and threatened of biodiversity hotspots (Colombo & Joly, 2010; Myers, Mittermeier, Mittermeier, da Fonseca, & Kent, 2000). Our mountaintop study site is home to many endemic species. Humans have exploited the Atlantic forest for 500 years resulting in a highly fragmented landscape (Joly, Metzger, & Tabarelli, 2014) that reduces possibilities for species migration. Hence, a greater understanding of forests in this region is of great interest given their high threat level. We focus here on determining and understanding interspecific differences in leaf temperatures caused by differences in leaf traits. Our approach aims to begin to reveal whether or not trees are likely to be able to cope with future conditions, and the extent to which species identity is likely to be important. This is a step towards an understanding of the resilience of tropical trees and is part of a broader effort to assess the effects of stressors on remaining Atlantic forests and their ability to recover.

We used a narrow canopy tower to access leaves of three trees each of different species (*Alchornea triplinervia* [Spreng.] Mull. Arg. [Euphorbiaceae], *Miconia cabussu* Hoehne [Melastomataceae], and *Guapira opposita* [Vell.] Reitz [Nyctaginaceae], hereafter referred to by genus only). We monitored leaf temperature and microclimate relevant to leaf energy balance over a period of 10 days and quantified the stomatal behaviour and structural leaf traits of the sample trees. With this dataset, we aim to answer the following questions:

- What are the current patterns of leaf temperature of the Atlantic forest species *Alchornea*, *Miconia*, and *Guapira* under fluctuating microclimatic conditions?
- Are there differences in leaf thermoregulation between the species?
- To what extent do leaf traits (width, stomatal conductance) and microclimate (radiation, *T*_A_, *D*, wind speed) determine leaf temperatures?

## 2 MATERIALS AND METHODS

### 2.1 Study site

The field study was carried out in the Serra do Mar State Park, São Paulo, Brazil. The park is home to the largest contiguous patch of Atlantic forest remaining, running along a steep coastal mountain range. The study site (23.3254S, 45.0938W) is located within a 1‐ha permanent plot at 1,000‐m elevation. The vegetation is mid‐successional secondary forest, regenerating from clear felling for charcoal before the establishment of the park in 1977 (Marchiori, Rocha, Tamashiro, & Aidar, 2016). The forest is classified as montane moist dense forest (Vieira et al., 2011), mean annual precipitation is 2,300 mm with a dry season in July and August, mean annual temperature is 17 °C (Joly et al., 2012), and fog occurs frequently (Rosado, Oliveira, & Aidar, 2010). Canopy height of emergent trees reaches 30 m. Data collection was carried out between October 1, 2016 and October 10, 2016.

### 2.2 Microclimate measurements

**Figure 1:**
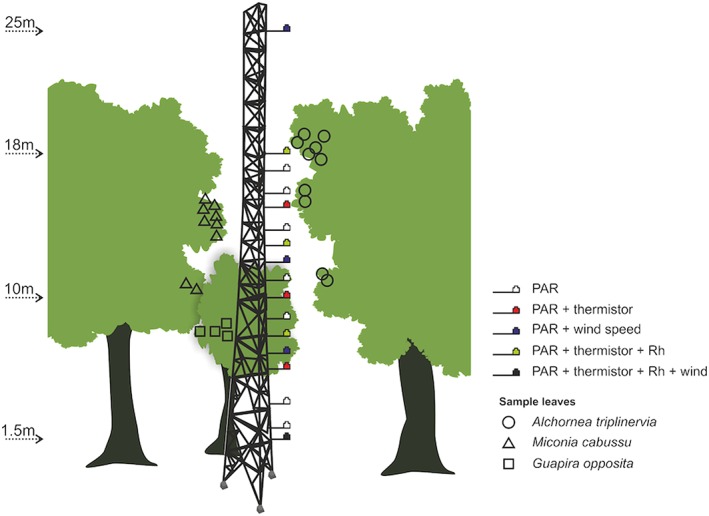
Schematic of field data collection showing positions of microclimate measurements and leaves sampled for temperature. PAR = photosynthetically active radiation

A narrow 27‐m‐high tower was used for access to the canopy and microclimate measurements (*T*_A_, photosynthetically active radiation [PAR], relative humidity [*h*], and wind speed [*U*]) were collected to detail the microclimate vertical profile (Figure 1). As the tower is just 30 cm wide and tree branches are within arms reach of the tower (see Figure S2d), we consider that the presence of the tower likely has only minimal influence on the microclimate of the sampled leaves. From 18 m above the ground, at the height of the highest leaves adjacent to the tower, 16 PAR sensors were suspended from the tower at *c*. 1‐m intervals, with an additional sensor positioned at 25 m above the ground. Sensors were made following Fielder and Comeau (2000) using gallium arsine phosphide photodiodes (G1118, Hamamatsu, Japan) and calibrated against a LI‐COR 190 quantum sensor (LI‐COR Inc., Nebraska, U.S.A.). PAR sensors were positioned on plastic supports in the horizontal plane. In addition, seven thermistors (107, Campbell Scientific, Utah, U.S.A.) to measure *T*_A_ were deployed in radiation screens spread through the vertical profile (heights 1.5, 5, 7.5, 10, 12.5, 15, and 18 m; Figure 1). PAR and *T*_A_ data were measured and recorded at 10‐s intervals using two CR800 data loggers with AM 16/32 multiplexers (Campbell Scientific, Utah, U.S.A.). Four data‐logging *h* sensors (RHT10, Extech, Massachusetts, U.S.A.) measured and recorded at 1‐min intervals at heights 2, 8, 12.5, and 18 m. Four sonic anemometers (Sonicwind) measured *U* every 0.5 s at heights of 1.5, 6.5, 11.5, and 25 m, and 10‐s averages were produced for each height. *U* for leaves positioned above 11.5‐m height was linearly interpolated between the 25‐ and 11.5‐m measurement.

Vapour pressure deficit (*D*) was calculated from *T*_A_ and *h* (Campbell & Norman, 1998),

(1) $$D=e_{\mathrm{sat}}\cdot \left(1-h\right),$$

(2) $$e_{\mathrm{sat}}=a\cdot \exp\cdot \left(\frac{bT_{A}}{T_{A}+c}\right),$$

where *T*_A_ is in °C, *h* is relative humidity (as a proportion), *e*_sat_ is saturating vapour pressure in kPa, and *a*, *b*, and *c* are constants (*a* = 0.611, *b* = 17.502, *c* = 240.97).

Due to a sensor fault, *h* was available only from October 5, 2016 to October 10, 2016. To estimate *D* within the profile for measurement days prior to this, we estimated *h* within the profile based on the observed relationship between *T*_A_ and *h* at the four measurement heights (*R* ^2^, .76–.87) from the available data collected over 6 days.

### 2.3 Sampled species

Both *Alchornea* and *Miconia* are overstorey species, whereas *Guapira* is found in the subcanopy (Guilherme, Morellato, & Assis, 2004), and the species are ranked second, fifth, and sixth, respectively, in terms of abundance in the plot (Marchiori et al., 2016). All species are early successional (Marchiori et al., 2016) and are found in nearby old‐growth forest. *Alchornea* and *Guapira* are present at lower elevations in the park (Joly et al., 2012; Rosado, Oliveira, Joly, Aidar, & Burgess, 2012). The species cover a range of leaf sizes; *Guapira* has the smallest leaves (notophyll), followed by *Alchornea* (mesophyll), with the largest leaves for *Miconia* (platyphyll).

### 2.4 Leaf temperature measurements

To measure leaf‐to‐air temperature differences (Δ*T*, also called *leaf temperature excess* in the literature) we followed the two‐junction thermocouple design of Singsaas and Sharkey (1998). This approach has the advantage of more accurately measuring Δ*T* than performing measurements of absolute *T*_L_ and *T*_A_ separately. Two long (15‐cm length) constantan fine wires (0.07‐mm diameter) were soldered to either end of a short (3‐cm length) copper fine wire (0.07‐mm diameter). These thermocouples produce a voltage proportional to the temperature difference between the junctions. Thermocouples were individually calibrated by inserting one junction into sand in a temperature controlled dry bath (TCBD‐02, Cleaver Scientific, U.K.) with the second junction in the air *c*. 2 cm above the bath. The temperature of the bath and the air were each measured with two thermistors. Four different temperature differences between the bath and air were produced (~0, 5, 10, and 12 °C). Data for the calibration were selected during periods with a constant dry bath temperature (i.e., excluding periods when the bath was heating up or cooling down).

Between September 30, 2016 and October 3, 2016 thermocouples were installed on 10 *Alchornea* leaves, 9 *Miconia* leaves, and 4 *Guapira* leaves. Selected leaves were fully expanded and mature, but not senescent, within reach from the canopy tower, and spread through the vertical profile (Figure 1). The thermocouple junction to measure leaf temperature was secured to the abaxial mesophyll surface (avoiding any large veins) near to the midrib using surgical tape (Transpore, 3M, Minnesota, U.S.A.). The second junction was suspended in the air *c*. 2 cm below the leaf. Additional cabling was cable tied to a twig near to the leaf (or the petiole in the case of *Miconia*) and to the tower. This attachment procedure enabled the majority of thermocouples to remain attached to leaves during wind and rain (see Figure S2 for photographs of the equipment installation). The petioles of two leaves, both of *Miconia*, snapped during the monitoring period. Table S1 gives details of the sampled leaves. Δ*T* was measured and recorded at 10‐s intervals using a CR800 data logger with AM 16/32 multiplexer (Campbell Scientific, Utah, U.S.A.) until October 11, 2016.

### 2.5 Spot measurements

In addition to continuous measurements of Δ*T* from the thermocouples, instantaneous spot measurements were made of leaf temperature using an infrared (IR) thermometer (62MAX+, Fluke, Washington, U.S.A.) on the adaxial and abaxial leaf surfaces, PAR at the adaxial leaf surface (accounting for leaf angle and orientation) with a quantum sensor (LightScout, Spectrum Technologies, Illinois, U.S.A.), and *g*_s_ using a porometer (SC‐1, Decagon Devices, Washington, U.S.A.). These measurements were made in order to (a) validate the thermocouple data against an independent *T*_*L*_ measurement, (b) compare PAR received at the leaf surface with that measured from the tower, and (c) investigate variation in *g*_s_ between species and over time. Spot measurements were collected during daylight hours throughout the day on 6 days between and October 4, 2016 and October 10, 2016. Stomatal conductance measurements could only be performed when the leaf surface was dry. Hence, fewer spot measurements of *g*_s_ were collected (213 in total, on average *c*. two measurements per leaf per day) compared with leaf temperature on adaxial surface (785 in total, on average *c*. six measurements per leaf per day), leaf temperature on abaxial surface (398 in total, on average *c*. three measurements per leaf per day), and PAR (350 in total, on average *c*. three measurements per leaf per day). Measurements of *g*_s_ with the SC‐1 porometer are completed in 30 s, and as the response of stomata to a change in the environment is on the order of minutes (e.g., Vialet‐Chabrand et al., 2017), we assume that the leaf *g*_s_ will not have changed due to the altered microclimate of the porometer chamber within the measurement interval. Leaf angle (angle to the horizontal) was measured sporadically (minimum of five measurements per leaf) using a clinometer (Suunto, Finland). No spot measurements were carried out during the night.

### 2.6 Leaf trait measurements

All sampled leaves were collected and stored in moist plastic bags for 24 hr before fully rehydrating and measuring structural traits in the laboratory at the Instituto de Botânica, São Paulo. Measurements were performed of leaf thickness (mm) with a digital calliper, leaf area (cm^2^) with leaf area meter (LI‐3100, LI‐COR, Nebraska, U.S.A.), leaf mass (g), leaf width (cm), and leaf length (cm). Petioles were removed before measurements. Subsequently, leaves were dried at 70 °C and dry‐weight measured. These measurements were used to calculate LMA (g/m^2^) and LDMC (g/g). For *Guapira*, the sample size for leaf traits was 6 (rather than 4 as for leaf temperature).

### 2.7 Leaf energy balance

With input of measured microclimate, stomatal conductance and leaf width the leaf energy balance (Equation (3), Jones, 1992) can be estimated to predict the leaf‐to‐air temperature difference (Δ*T*_e_, °C). It is important to note that the leaf energy balance assumes no leaf heat storage and that the leaf energy balance is considered to be in a steady state. Δ*T*_e_ was estimated from spot measurements to test if Δ*T*_e_ matched observations of Δ*T* when leaf surface PAR and *g*_s_ were directly measured, and from the continuous microclimate data with *g*_s_ estimated from the observed species‐specific relationships between *g*_s_ and *D* in order to assess the influence of microclimate and leaf specific variables on leaf temperatures using a large dataset. As the *Guapira* leaves were not exposed to a large range of microclimates due to their position in the understorey, we only consider Δ*T*_e_ of *Miconia* and *Alchornea* in the latter analysis.

(3) $${\mathrm{\Delta T}}_{e}=T_{L}-T_{A}=\frac{r_{b,\mathrm{HR}}\left(r_{b,W}+r_{l,W}\right)\gamma R_{\mathrm{ni}}}{\rho_{a}c_{\mathrm{pa}}\left[\gamma \left(r_{b,W}+r_{l,W}\right)+sr_{b,\mathrm{HR}}\right]}-\frac{r_{b,\mathrm{HR}}D}{\gamma \left(r_{b,W}+r_{l,W}\right)+sr_{b,\mathrm{HR}}},$$

where *T*_L_ and *T*_A_ are the leaf and air temperatures, respectively (°C), *R*_ni_ is the net isotropic radiation (W/m^2^, assuming the sky temperature is equal to *T*_*A*_ measured at the nearest *T*_A_ sensor to the leaf and sky emissivity of 0.97), γ is the psychrometric constant (Pa/K), *r*_b,HR_ is the boundary layer resistance to heat and radiation and *r*_b,W_ and *r*_l,W_ are the boundary layer and leaf resistances to water, respectively (all resistances in seconds per metre), *ρ*_a_ is the density of dry air (kg/m^3^), *c*_pa_ is the specific heat capacity of dry air (1,012 J·kg^−1^·K^−1^), *s* is the slope of relationship between temperature and saturated vapour pressure evaluated at *T*_A_, and *D* is the vapour pressure deficit (Pa).

Leaf traits (*g*_s_ and leaf width) are included in Equation (3) through the leaf and boundary layer resistances. Leaf resistance to water, *r*_l,W_, is the inverse of *g*_s_. Boundary layer conductance to heat or water, *g*_b,H_, which is included in the determination of both *r*_b,HR_ and *r*_b,W_, which are both used in Equation (3), is dependent on leaf width (*W*, m) and wind speed (*U*, m/s)

(4) gb,H=0.0105UW0.5.

Further details on the estimation of leaf energy balance are given in Appendix S1.

The thermal time constant (τ, s) was defined following Michaletz et al. (2016) as

(5) $$\tau =\varphi \cdot \mathrm{LMA}\cdot \left(\frac{c_{\mathrm{pw}}}{\text{LDMC}\cdot H}+\frac{c_{\mathrm{pd}}-c_{\mathrm{pw}}}{H}\right),$$

where *φ*, the ratio of projected to total leaf area, is 0.5 for flat leaves; LMA is in kilograms per square metre; *c*_pw_ is the specific heat capacity of water (4,181 J·kg^−1^·K^−1^); and *c*_pd_ is the specific heat capacity of dry leaf matter (J·kg^−1^·K^−1^). *c*_*pd*_ varies by species and, here, we use 2,814 J·kg^−1^·K^−1^, the mean of seven tropical tree species from Jayalakshmy and Philip (2010). *H* is a heat transfer coefficient (W·m^−2^·K^−1^) accounting for convection, radiation, and transpiration (Michaletz et al., 2016).

(6) $$H=\rho_{a}c_{\mathrm{pa}}\left(g_{b,H}+g_{b,R}+g_{s}s/\gamma \right),$$

where *g*_b,H_ and *g*_b,R_ are the boundary layer conductance to heat and radiation, respectively (both are in metres per second; see Appendix S1). τ varies over time due to its dependence on *g*_s_ and boundary layer resistance and was estimated from spot measurements.

### 2.8 Leaf boundary layer resistance

Initial estimations of the leaf energy balance using Equation (3) showed that when Δ*T*_e_ was evaluated at low wind speeds (<0.5 m/s), the values were overestimated compared with the observed Δ*T*. Using Equations (4) and S5 to estimate the boundary layer resistance to water (*r*_b,W_), there is a steep increase in *r*_b,W_ below wind speeds of 0.5 m/s (Figure S3). To test if these high resistances were supported by the data, we solved the leaf energy balance equation for *r*_b,W_ and estimated *r*_b,W_ using the observations of Δ*T* (see Appendix S2). Plotted against wind speed, the estimated *r*_b,W_ was lower than predicted by Equations (4) and S5 at low wind speeds (Figure S3). Hence, we reparameterized constants from Equation (4) using the *r*_b,W_ estimated from the leaf energy balance and observed wind speed and leaf width (see Appendix S2). Parameter estimation was performed separately for *Miconia* and *Alchornea* (there was not sufficient data for parameter estimation of *Guapira*) using non‐linear least squares (R function nls).

In order to have accurate estimates of *r*_b,W_ from the energy balance, it is essential that all microclimate inputs are correct. PAR was measured at various points from the tower (Figure 1). Examination of the spot measurement data showed that PAR measured by the nearest sensor suspended from the tower (maximum 1‐m distance from leaf) occasionally strongly overestimated or underestimated leaf surface PAR (Figure S4) as they are not measured at precisely the same location, angle, or orientation, and PAR shows high spatial variability. To select only data where PAR measured from the tower appropriately represented PAR at the leaf surface, the daytime data were split into 20‐min periods and Δ*T*_e_ estimated for every 10‐s datapoint. Linear regression was then used to identify periods where Δ*T*_e_ matched measured Δ*T*, selecting only periods where the slope of the relationship between Δ*T*_e_ and Δ*T* was 1 ± 0.3 and the intercept was ±2 °C. On the basis of this selection procedure, we identified 20% of the dataset (*c*. 150,000 data points) considered to have representative PAR measurements. This approach does not entirely eliminate noise from the dataset as within the 20‐min period, there can still be some erroneous data points.

### 2.9 Data analysis

Linear mixed‐effects models with leaf as a random factor were used for all statistical analyses including repeated measurements of the same leaf using the R package nlme (Pinheiro, Bates, DebRoy, Sarkar, & Core Team, 2017). *R* ^2^ for mixed‐effects models are given using either the marginal pseudo *R* ^2^ that accounts for fixed factors only or conditional pseudo *R* ^2^ (Nakagawa & Schielzeth, 2013). The marginal pseudo *R* ^2^ is used unless otherwise stated, and *R* ^2^ values were calculated using the function provided in the R package MuMIn (Bartoń, 2016). Statistical analyses comparing between species using single values for each leaf used analysis of variance for three species comparisons and *t* test for two species comparisons.

Relationships between *g*_s_ and *D* were analysed for each species using a linear mixed‐effects model with leaf as a random factor. The relationships produced were used to estimate a time series of *g*_s_ for each leaf based on *D*. The intercept of the *g*_s_–*D* relationship was thus leaf specific and the slope species specific.

To compare leaf temperatures under comparable microclimate conditions data were first selected for 20‐min periods where Δ*T*_e_ matched measured Δ*T* to ensure that microclimate variables are representative of the leaf surface, as for leaf boundary layer resistance (see above) but using the species‐level parameterization of *r*_b,W_ to estimate Δ*T*_e_. The selected dataset was then subsetted according to the microclimate (PAR, *T*_A_, and *U*) for each leaf. We produced subsets of Δ*T* under low PAR and *T*_A_ (PAR, 50–150 μmol·m^−2^·s^−1^; *T*_A_, 13–15 °C), medium PAR and *T*_A_ (PAR, 50–150 μmol·m^−2^·s^−1^; *T*_A_, 13–15 °C), high PAR and *T*_A_ (PAR, 1,000–1,300 μmol·m^−2^·s^−1^; *T*_a_, 18–20 °C), and very high PAR (PAR, 1,600–1,900 μmol·m^−2^·s^−1^; *T*_*A*_, 18–20 °C), all at wind speed of 0.5–1.5 m/s. Differences in Δ*T* between species for each microclimate were evaluated with linear mixed‐effects models with leaf as a random factor.

## 3 RESULTS

### 3.1 Validation of thermocouple data

*T*_L_ based on Δ*T* measured with thermocouples and *T*_A_ measured with thermistors (*T*_L_ = Δ*T* + *T*_A_) was highly correlated with *T*_L_ as measured by the IR thermometer (Pearson's correlation coefficient for each leaf .60–.99, where *T*_L_ was measured with IR thermometer on the abaxial leaf surface). The slopes of linear regression lines forced through 0 were significantly different from 1 for only three leaves, where the thermocouples slightly underestimated *T*_L_ by up to 9% (Figure S5). Overall, the close agreement between the two measurement methods gives confidence in the thermocouple data.

### 3.2 Microclimate during the monitoring period

**Figure 2:**
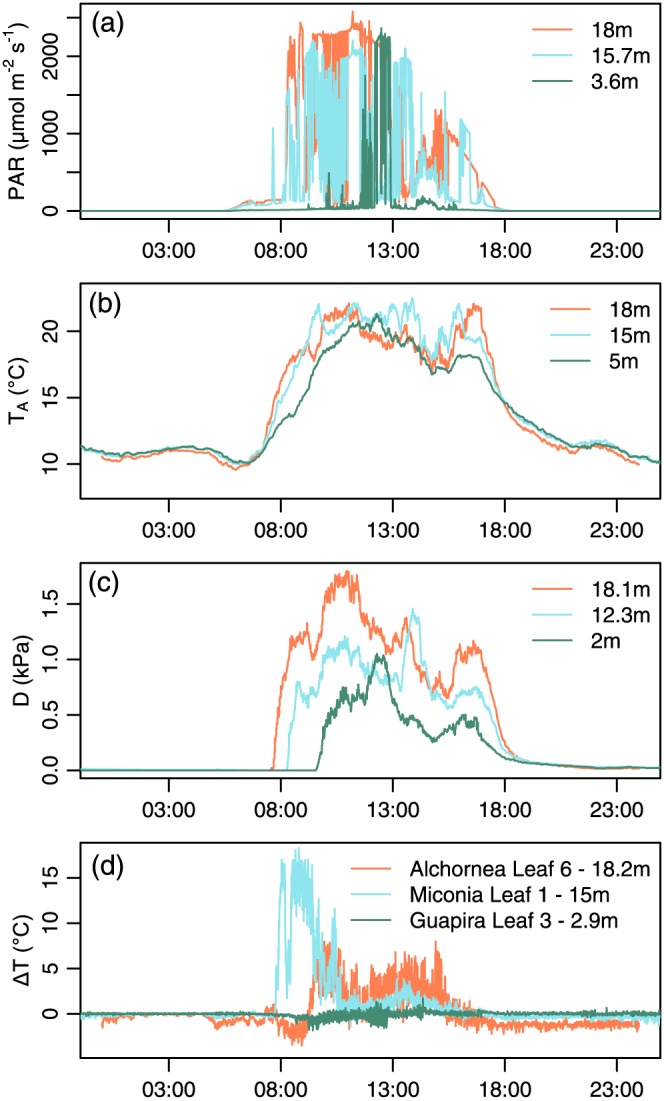
Time series of microclimate and leaf‐to‐air temperature difference on October 8, 2016. (a) Photosynthetically active radiation (PAR), (b) air temperature, (c) vapour pressure deficit, and (d) leaf‐to‐air temperature difference for leaves of *Alchornea* (A6), *Miconia* (M1), and *Guapira* (G3). Colours refer to measurement heights [Colour figure can be viewed at http://wileyonlinelibrary.com]

Microclimate during the monitoring period is shown in Figure S6 and for a single sunny day in Figure 2. The first 7 days (October 1 to October 7) were predominately overcast with low PAR, high *h*, and low *D*, with some sunny periods on October 6 and October 7. Subsequently, 2 days (October 8 to October 9) had longer bright periods. The final day of data collection (October 10) was again overcast. Throughout the period, lower canopy levels received substantially less PAR and experienced lower *D*. However, on sunny days, high PAR levels and higher *D* extended throughout the vertical profile (e.g., October 8, 2016; Figure 2). Mean daytime *T*_A_ at the top of the canopy was 15.0 °C, with a maximum *T*_A_ of 22.1 °C recorded on October 8, 2016. Mean night‐time *T*_A_ was 12.4 °C and was lowest preceding sunny days. Mean wind speed above the canopy (at 25 m) was 1.0 ± 0.7 m/s and 0.26 ± 0.18 m/s within the canopy (averaged over all sample heights 1.5, 6.5, and 11.5 m).

### 3.3 Patterns of leaf temperature

Leaves occasionally reached much higher temperatures than the ambient air, over 10 °C above *T*_A_. The maximum *T*_L_ for each leaf observed over the monitoring period ranged from 22.5 to 37.2 °C and was above 35 °C for five of the 23 leaves. *Miconia* leaves had significantly higher maximum temperatures than *Alchornea* leaves (Table 1). Daytime mean *T*_L_ showed less variation than maximum *T*_L_ between leaves and species (Table 1, Figure S7). Leaves of *Guapira*, which were all at the bottom of the canopy, had lower maximum and mean *T*_L_ (not tested for significance due to low sample size). Distributions of *T*_L_ show positive skew (Figure S7), which was significantly higher for leaves of *Miconia* than *Alchornea* (Table 1), showing that they reached high temperatures more often than *Alchornea*. During daylight hours Δ*T* was positive for leaves of *Alchornea* and *Miconia* and was close to 0 for all *Guapira* leaves (Figure S8, Table 1). The maximum Δ*T* observed was 18.3 °C, recorded from *Miconia* leaf M1. As for *T*_L_ the distributions of Δ*T* were positively skewed, with significantly higher skewness for *Miconia* than *Alchornea* (Table 1). Minimum daytime Δ*T* was significantly lower for *Alchornea* than *Miconia* leaves (Table 1). Night‐time Δ*T* was typically negative but close to 0 and was significantly lower for *Alchornea* than *Miconia* (Table 1).

**Table 1:** Leaf temperature variables for three species

	Mean ± *SD*			
	*Alchornea* (*n* = 10)	*Miconia* (*n* = 9)	*Guapira* (*n* = 4)	*p* *
Daytime minimum *T*_L_ (°C)	7.71 ± 0.63	8.51 ± 1.55	8.13 ± 0.59	.18
Daytime mean *T*_L_ (°C)	15.98 ± 0.47	16.14 ± 0.72	14.6 ± 0.01	.6
Daytime maximum *T*_L_ (°C)	30.56 ± 3.6	34.63 ± 2.63	23.33 ± 1.22	.012
Daytime *T*_L_ skewness	0.84 ± 0.43	1.38 ± 0.27	0.56 ± 0.27	.005
Daytime minimum Δ*T* (°C)	−3.72 ± 1.36	−2.16 ± 0.75	−5.07 ± 2.50	.007
Daytime mean Δ*T* (°C)	0.79 ± 0.40	0.84 ± 0.43	0.007 ± 0.001	.8
Daytime maximum Δ*T* (°C)	11.27 ± 4.15	14.23 ± 2.71	3.28 ± 1.08	.08
Daytime Δ*T* skewness	2.51 ± 1.32	4.56 ± 1.63	0.73 ± 5.86	.009
Night‐time mean Δ*T* (°C)	−0.13 ± 0.07	−0.06 ± 0.03	−0.008 ± 0.01	.02

*Note*. *n* = number of leaves measured for each tree.

* *p* value from *t* tests comparing *Alchornea* and *Miconia*.

**Figure 3:**
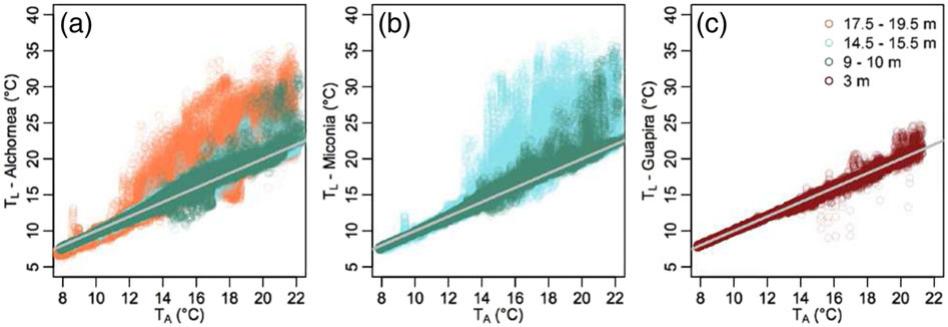
Leaf (*T*_L_) and air (*T*_A_) temperatures measured over 10 days for (a) *Alchornea* (10 leaves), (b) *Miconia* (10 leaves), and (c) *Guapira* (4 leaves). Colour denotes leaf height. Grey line: *y* = *x*. Each data point is a single measure of a single leaf taken from the thermocouple time series recorded every 10 s [Colour figure can be viewed at http://wileyonlinelibrary.com]

*T*_A_ set a rough minimum bound on *T*_*L*_ (Figure 3), with many excursions above *T*_A_ due to high radiation (see Section 3.6) and a small number of excursions below *T*_A_, likely occurring when leaf surfaces were wet during/after rain or in fog. *T*_L_ excursions above *T*_A_ occurred more often for leaves positioned higher in the canopy.

### 3.4 Leaf temperatures under specific microclimates

**Figure 4:**
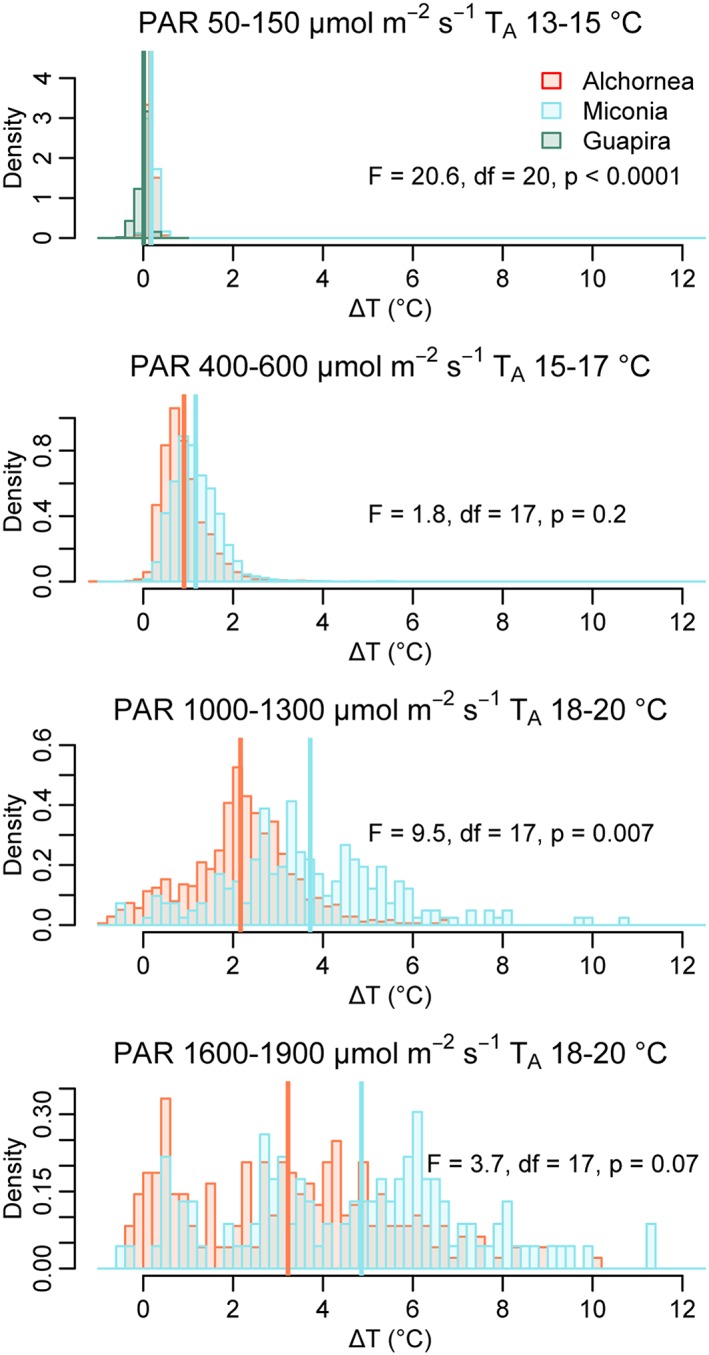
I notice that I made an error in the box colour for the legend of this figure and here attach a replacement with the correct colouring.Leaf to air temperature difference (Δ*I notice that I made an error in the box colour for the legend of this figure and here attach a replacement with the correct colouring.T*I notice that I made an error in the box colour for the legend of this figure and here attach a replacement with the correct colouring.) variation between species and microclimatic conditions. Vertical lines show the mean value for the species. Significance values are shown testing for differences between species (linear mixed‐effects model with leaf as a random factor) under four different microclimates. PAR = photosynthetically active radiation; *T*_A_ = air temperature [Colour figure can be viewed at http://wileyonlinelibrary.com]

We compared leaf temperatures under specific microclimatic conditions. Under low light and temperature conditions, leaves of *Guapira* were significantly colder than those of *Alchornea* and *Miconia* (Figure 4a); there was no significant difference in Δ*T* between the latter two species. Under medium light and temperature conditions, there again was no significant difference in Δ*T* between *Alchornea* and *Miconia* (Figure 4b); *Guapira* leaves did not experience these or brighter light conditions due to their position in the understory. Under high light and temperature conditions, Δ*T* was significantly higher for *Miconia* than *Alchornea* (Figure 4c). Under the highest light conditions analysed, Δ*T* was again higher for *Miconia* than *Alchornea*; however, the difference was not quite significant (Figure 4d).

### 3.5 Thermal trait variation between species

**Figure 5:**
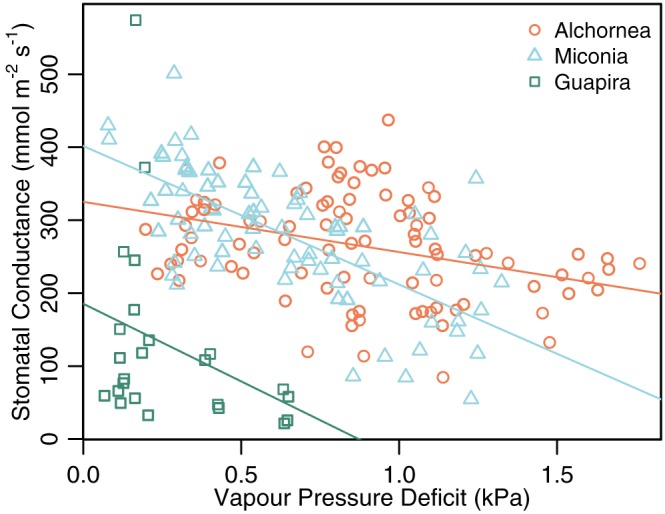
Relationship between stomatal conductance and vapour pressure deficit, and variation between species. Equations for each species—Alchornea: *g*_s_ = 325.1 (±22.4) − 68.8 (±22.6) *D*; *Guapira*: *g*_s_ = 185.4 (±36.4) − 212.7 (±22.6) *D*; *Miconia*: 401.6 (±31.0) − 189.8 (±35.4) *D* (errors are standard) [Colour figure can be viewed at http://wileyonlinelibrary.com]

Stomatal conductance (*g*_s_) significantly declined with increasing *D*, and the relation varied significantly between species (Figure 5, Table 2). At low *D*, *g*_s_ was highest for *Miconia* and lowest for *Guapira*. *Miconia* showed a significantly stronger negative relationship between *g*_s_ and *D* than *Alchornea*; hence, at higher values of *D*, *Miconia* leaves had lower *g*_s_. Conditional *R* ^2^ for the overall mixed model including the random factor for leaf was 0.49.

**Table 2:** Analysis of variance table for the linear mixed‐effects model of *D*, species, and their interaction on *g*_s_. Leaf is included as random intercept

	Numerator *df*	Denominator *df*	*F*	*p*
Intercept	1	167	1097.0	<.0001
*D*	1	167	18.7	<.0001
Species	2	20	40.0	<.0001
*D*: Species interaction	2	167	6.6	.0018

**Figure 6:**
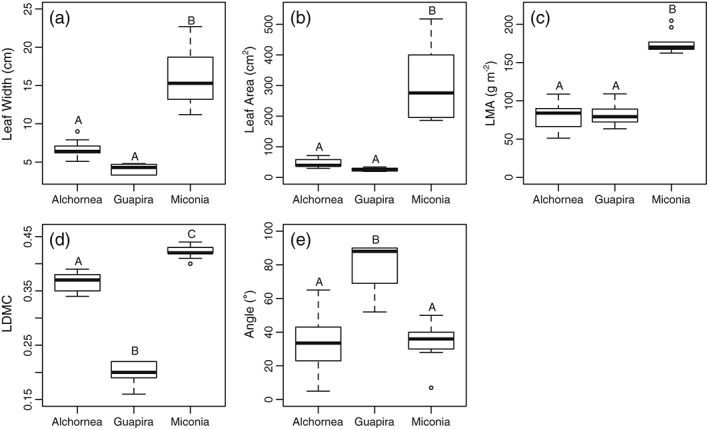
Variation in leaf structural traits between species. Letters represent significantly different groups calculated using analysis of variance and Tukey post hoc tests

Structural leaf traits with importance for thermoregulation also varied between species (Table S1, Figure 6). *Miconia* leaves were significantly wider, larger, and had higher LMA than both *Alchornea* and *Guapira* (Figure 6a–c). LDMC significantly differed between all species and was highest for *Miconia* (0.42 ± 0.013 g/g) followed by *Alchornea* (0.37 ± 0.016 g/g), and finally *Guapira* (0.20 ± 0.022 g/g; analysis of variance, *F* = 38.8, *p* < .0001, and Tukey post hoc test).

**Figure 7:**
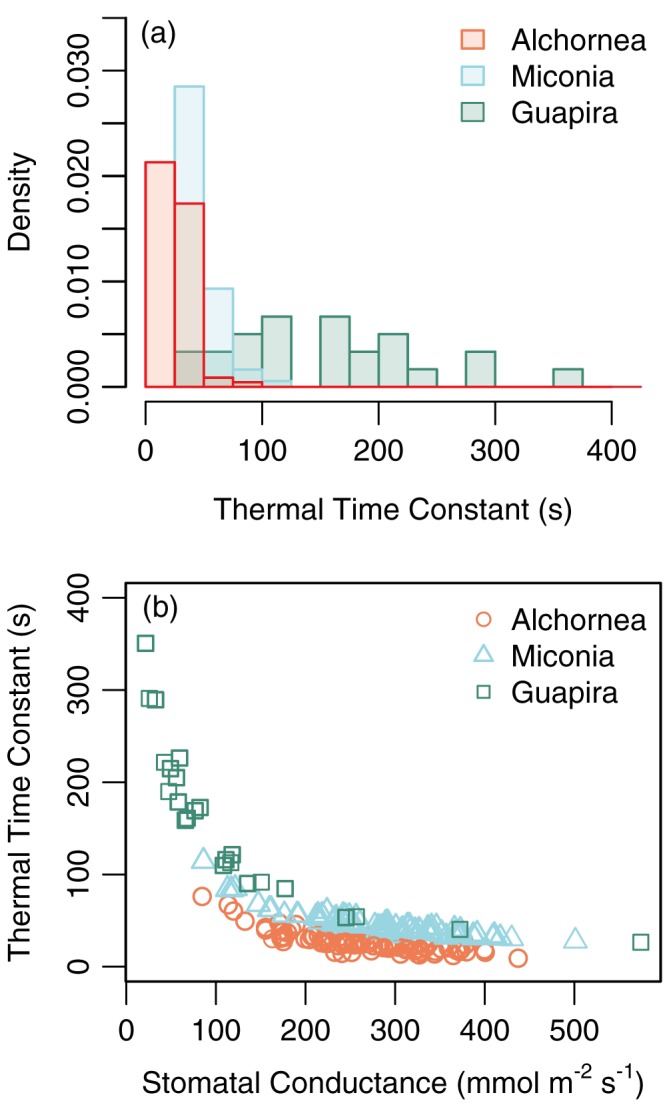
(a) Distributions of thermal time constants estimated for each species and (b) the relationship between stomatal conductance and the thermal time constant [Colour figure can be viewed at http://wileyonlinelibrary.com]

The thermal time constant (τ) ranged over two orders of magnitude from 9 to 350 s (Figure 7a) and varied significantly between species (linear mixed effects model, *F* = 48.1, *df* = 20, *p* < .0001). τ for *Guapira* were significantly longer and more varied (mean ± *SD* 155.4 ± 84.0) than both *Alchornea* (mean ± *SD* 276.5 ± 11.1) and *Miconia* (mean ± *SD* 46.4 ± 14.4). τ decreased with increasing *g*_s_ and was particularly high under very low *g*_s_ (Figure 7b). For a given *g*_s_, τ increased in the order *Alchornea* < *Miconia* < *Guapira* (Figure 7b). These differences were driven by the leaf structural traits LMA and LDMC (Figure S9). When estimated using a fixed LMA value the differences between *Alchornea* and *Miconia* are lost (Figure S9b) showing that the higher LMA of *Miconia* increases τ. When estimated using a fixed LDMC value the *Guapira* values collapse into line with *Alchornea* (the two species have similar LMA; Figure S9c) showing that the lower LDMC of *Guapira* increases τ.

### 3.6 Leaf energy balance and drivers of ΔT and T_L_

**Figure 8:**
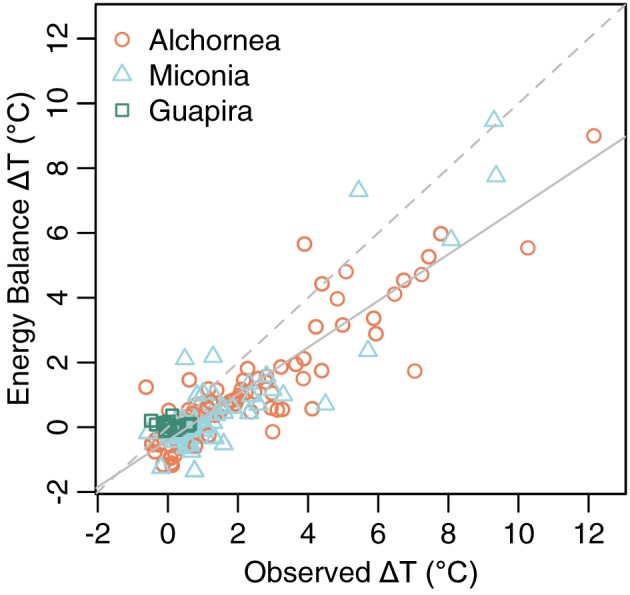
Leaf to air temperature difference (Δ*T*) from observations and energy balance estimations. Grey dash: *y* = *x* line; grey solid: linear regression line for all data; regression slope = 0.72 ± 0.03 SE; intercept = −0.41 ± 0.07 SE; *F* = 725.8; *df* = 185; *p* < .0001; *R* ^2^ = .80. SE = standard error [Colour figure can be viewed at http://wileyonlinelibrary.com]

**Figure 9:**
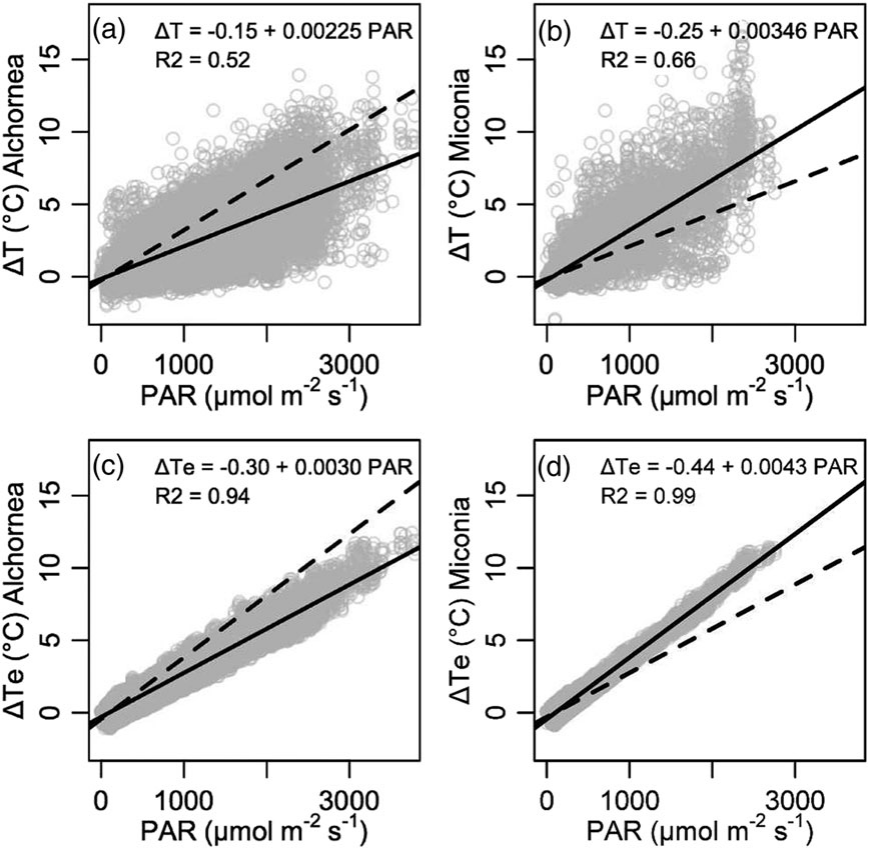
(a, b) Relationships between PAR and observed Δ*T* and (c, d) estimated Δ*T*_e_ for (a, c) *Alchornea* and (b, d) *Miconia*. Solid line—modelled relationship for the plotted species. Dash line—modelled relationship for the alternative species. Statistical models are linear mixed‐effects model with leaf as a random factor. *R* ^2^ is the marginal pseudo *R* ^2^. To account for uneven sampling with respect to PAR data was subsampled for 1,000 points in 250 μmol·m^−2^·s^−1^ bins for points below 1,000 μmol·m^−2^·s^−1^

Leaf‐to‐air temperature difference estimated from leaf energy balance (Δ*T*_e_) using the spot measurements matched observed Δ*T* well but with some underestimation at higher Δ*T* (Figure 8), showing that our data adequately parameterized the leaf energy balance for instances when leaf surface PAR and *g*_s_ were measured. To investigate the drivers of Δ*T* with the larger dataset of continuous Δ*T* and microclimate measurements, the dataset was restricted to periods where predicted Δ*T*_e_ matched observed Δ*T*, as for the analysis of Δ*T* under specific microclimate conditions. This is to ensure we are using appropriate values of PAR, which was not measured at the leaf surface in the continuous dataset. Both observed Δ*T* and Δ*T*_e_ increase with PAR (Figure 9), a pattern repeated when *T*_L_ and *T*_Le_ (leaf temperature estimated from energy balance) were analysed (Figure S13). The slope of the relationship between leaf temperature variables and PAR were different between *Miconia* and *Alchornea*, where *Miconia* has higher Δ*T* and *T*_L_ for a given PAR (Figure 9 and S13). Although the absolute values of Δ*T*_e_ and *T*_Le_ are somewhat higher than the observations, the differences between the species are maintained in the energy balance estimations. Relationships between Δ*T* and Δ*T*_e_ and other microclimate variables (*T*_A_, *D*, and *U*) were much weaker than for PAR with all *R* ^2^ values below 0.3 (Figures S10–S12), whereas *T*_L_ and *T*_Le_ were strongly related to *T*_A_ and *D* with *R* ^2^ values above 0.7 (Figures S14–S16).

To determine what causes the differences between Δ*T* of *Miconia* and *Alchornea*, we applied traits (leaf width and stomatal conductance strategy) of *Miconia* sequentially to *Alchornea* and re‐estimated Δ*T*_e_ using the observed microclimate data. As shown in Figure S17, applying the larger leaf width of *Miconia* acts to increase the *Alchornea* Δ*T*_e_ for a given PAR, almost to the extent that it matches the high Δ*T*_e_ of *Miconia*. If the higher intercept of the *g*_*s*_–*D* relationship for *Miconia* is applied, the *Alchornea* Δ*T*_e_ for a given PAR decreases. In contrast, if the steeper *g*_*s*_–*D* slope for *Miconia* is applied, the *Alchornea* Δ*T*_e_ for a given PAR increases. The effect is not as strong as the effect of leaf width (Figure S17). Applying both the *Miconia* intercept and slope results in an intermediate *Alchornea* Δ*T*_e_ for a given PAR, slightly higher than for *Alchornea* with its original parameterization. If all *Miconia* traits are applied (leaf width and stomatal conductance strategy) *Alchornea* Δ*T*_e_ for a given PAR increases to a greater extent than for any trait alone and even exceeds the Δ*T*_e_ of *Miconia*. This is likely due to the higher *D* that the highest six *Alchornea* leaves are exposed to due to their position above the *Miconia* leaves (Figure 1).

## 4 DISCUSSION

### 4.1 Current leaf temperature patterns and the role of microclimate

Despite the low *T*_A_ (maximum 22 °C) occurring during the study period, we observed leaf temperatures over 30 °C. Although few datasets are available on field‐measured leaf temperatures of tropical trees, the maximum Δ*T* we observed (18.3 °C) is somewhat higher than those previously reported (e.g., 13.9 °C for a range of Proteaceae species in Australia [Leigh, Sevanto, Close, & Nicotra, 2017], 10 °C for five species in Panama [Rey‐Sanchez et al., 2016] and in the Amazon [Doughty & Goulden, 2008], 7 °C for three species in tropical China [Dong, Prentice, Harrison, Song, & Zhang, 2017]). This could be due to high sampling frequency used in this study (every 10 s) compared with others (every 2 min in Rey‐Sanchez et al., 2016, half hourly in Dong et al., 2017 and unspecified in Leigh et al., 2017) as at high frequency extreme values are more likely to be recorded, though Doughty and Goulden (2008) used a 1‐s sampling frequency. It could also be due to the light conditions and study species measured (discussed below). The highest *T*_L_ observed (37.2 °C) is less than those reported by others under higher ambient air temperatures (e.g., 45 °C—Doughty & Goulden, 2008; 48 °C—Slot et al., 2016, Krause et al., 2010). This work supports the view that ambient air temperatures cannot necessarily be used as a proxy for leaf temperature in physiological models as they are not necessarily equal (Michaletz et al., 2016; Rey‐Sanchez et al., 2016) and that vegetation models should be tested for their ability to reproduce patterns of Δ*T* (Dong et al., 2017).

The distributions of *T*_L_ and Δ*T* collected over the 10‐day period were significantly skewed (Figures S7 and S8 and Table 2). This is because under the predominant microclimatic conditions of relatively low PAR and *D*, Δ*T* is low (<1 °C) and *T*_L_ is similar to *T*_A_. However, due to fluctuating conditions—especially PAR, which alters rapidly with cloud movements and wind and varies with sun angle, leaf angle, and orientation—large increases in Δ*T* occur. The duration of high Δ*T* excursions depends on how long the microclimate is sustained. The extent of high Δ*T* excursions is important because during high leaf temperatures beyond the photosynthetic temperature optima primary productivity will reduce carbon gain and very high leaf temperatures can cause irreversible thermal damage (e.g., above 50–53 °C for a Panamanian tree species; Krause et al., 2010). Our data suggest that, at least during our measurement period, tree leaves at this Atlantic forest site are not approaching thresholds of irreversible damage but do reach temperatures known to affect thylakoid membrane structure (35 °C; Gounaris et al., 1983; Gounaris et al., 1984) and reduce electron transport rates (40 °C; Allakhverdiev et al., 2008) although critical temperatures of PSII activity are known to vary among species (O'Sullivan et al., 2017; Sastry & Barua, 2017). Although we do not know the photosynthetic temperature optima of these trees, it is likely that the higher leaf temperatures reached were supraoptimal for photosynthesis despite the low air temperatures.

Within the range of conditions during the study period, radiation was the most important microclimate variable for determining Δ*T* (Figure 9, Figures S10–S12). This has also been shown in other studies (e.g., Doughty & Goulden, 2008; Rey‐Sanchez et al., 2016) and is understood biophysically (Jones, 1992). For absolute *T*_L_, PAR, *T*_A_, and *D* were all important (Figures S13–S16), though the strength of the *D* effect is likely due to at least in part to its covariation with *T*_A_. Recent work has shown the occurrence of a *crossover T*_A_ at 25–28 °C (Dong et al., 2017; Michaletz et al., 2016). The crossover temperature is the *T*_A_ at which Δ*T* = 0 and above which Δ*T* is negative. We found no evidence of a crossover *T*_A_, likely due to the relatively low *T*_A_ during our study. The light levels observed in the study were high, occasionally exceeding 3,000 μmol·m^−2^·s^−1^. We consider the light levels recorded in the profile to be accurate as they were highly comparable with an independent dataset from a weather station mounted at 27 m on the same tower (Figure S18). The values are higher than the PAR observed in similar studies from lowland forests with typical maximum PAR of 2,000 μmol·m^−2^·s^−1^ (Doughty & Goulden, 2008; Rey‐Sanchez et al., 2016). Again, measurement frequency may be important here for recording extreme instantaneous values. In fact, this maximum quantity of PAR is equivalent to more radiation than the solar constant (incoming light at the top of the atmosphere, 1.353 kW/m^2^), which is possible in mountains when light is reflected from clouds (Stoutjesdijk & Barkman, 2014). Incoming radiation increases by 8% for every 1,000‐m increase in elevation (Blumthaler, Ambach, & Ellinger, 1997). Montane forests are therefore likely to experience higher maximum radiation loads than lowland forest, as has been measured at this site (Rosado, Joly, Burgess, Oliveira, & Aidar, 2016). Given the importance of radiation for *T*_L_, trees at high elevation may have greater risk of hitting damaging *T*_L_ thresholds if air temperatures increase with climate change. At this specific site, in additional to increased radiation, *D* also increases with elevation and trees show more conservative water use (Rosado et al., 2016), which will further influence leaf temperatures. Mountaintop species are already considered to be more greatly threatened than lowland species by increased temperatures as there is no cooler place for species to move to. The high radiation load increasing leaf temperatures may exacerbate this problem.

As microclimate is a key driver of leaf temperature, it is important to consider the vertical gradient in microclimate (Figure S6). We found that all microclimate variables displayed vertical gradients, especially during sunny days when the differences between the top and bottom of the canopy exceeded 5 °C *T*_A_, 2,200‐μmol·m^−2^·s^−1^ PAR and 1.3‐kPa *D*. The difference in *T*_A_ leads to a larger difference in *T*_L_ than the values of Δ*T* we typically found (Table 1). Although vertical gradients of PAR are often accounted for in vegetation models, often the gradients of other key variables are not considered, which would lead to error in quantification of leaf temperatures below the canopy top.

### 4.2 Differences in leaf thermoregulation between species

We found striking differences in leaf temperature patterns between species that were attributable to differing leaf traits. *Miconia* leaves more commonly experienced high Δ*T* excursions than *Alchornea*, with higher skew in *T*_L_ and Δ*T* distributions, higher maximum Δ*T*, and less negative minimum Δ*T* (Table 1). Leaf temperatures of *Miconia* were consistently higher than *Alchornea* when controlling for microclimate between measurements and significantly so during high light conditions (Figure 4). The differences increased with increasing thermal stress (higher PAR, *T*_A_, and *D*). The lack of significance at the highest PAR/*T*_A_ subset tested is likely due to low data availability and higher PAR measurement errors at high PAR. As PAR was not measured directly at the leaf surface, it was difficult to ensure correspondence between PAR as measured by the nearest sensor and received at the leaf surface; this is more problematic under direct light conditions where leaf angle, orientation, sun angle, and within‐canopy shading greatly impact leaf surface PAR. We recommend all studies of leaf temperature attempt to measure PAR at the leaf surface despite the higher efforts required.

The higher leaf temperatures displayed by *Miconia* can be accounted for by lower transpirational cooling due to two reasons. Firstly, the wider leaf width increases boundary layer resistance, which lowers the evaporation from stomatal pores. Secondly, *Miconia* leaves showed a strong negative relationship between *g*_s_ and *D*, which lowers transpiration under conditions of high thermal stress (as high *D* typically occurs concurrently with high PAR and *T*_A_). Using the leaf energy balance equation, we find that the physical difference in leaf width is the dominant factor in producing the variation in Δ*T* between *Miconia* and *Alchornea* (Figure S17). *Miconia* leaves get hotter than *Alchornea* leaves and, hence, may have a higher risk of thermal damage. However, this increased heating may come with a water use advantage, as, under high *D* conditions, transpiration rates per leaf area will be lower for *Miconia* than *Alchornea*. This could reduce the risk of xylem cavitation under water stress conditions. Differing thermoregulation strategies of trees likely arise in combination with trade‐offs in terms of water use.

The study species only showed differing relationships between PAR, and *T*_L_ and Δ*T*, with similar responses to other microclimatic variables (Figure 9, Figures S10–S16). This shows that it is the consequences for input solar energy that varies between the species, rather than differing mechanisms in response to *T*_A_. It is not to say that other microclimatic variables are not important for *T*_L_ or Δ*T* but that the response of *T*_L_ and Δ*T* to other variables is the same for the two species, at least under the measurement conditions.

Night‐time Δ*T* were consistently negative for all species. However, Δ*T* of *Alchornea* leaves were more negative than the other species (Table 1). The cause may be that many of the sampled *Alchornea* leaves were at the outer canopy, and therefore, heat radiation to space may be more effective for them due to the lack of obstacles (other leaves or canopies), resulting in greater cooling. Another factor may be that transpiration is maintained at night in this species more so than *Miconia* and *Guapira*. Observations from Rosado et al. (2012) do show night‐time transpiration occurring for *Alchornea* trees at this site, but *Alchornea* did not show higher transpiration than other measured species.

Leaf temperatures of the subcanopy *Guapira* tree were consistently similar to air temperatures and showed little variation (Table 1) likely due to the canopy position receiving very little light (Figure 1). However, when the data were subsetted for low PAR conditions only, leaves of *Guapira* still showed a lower Δ*T* than the two other species (Figure 4a). This could be due to the narrower leaf width of *Guapira* leaves (Figure 6), though the width is not significantly different from *Alchornea*. It could also be due to the unusual leaf angles displayed by the *Guapira* leaves that were hanging near vertically (Table S1, Figure 6e), which would limit the amount of light received and result in over estimates of the light environment from using a horizontally orientated sensor. Another potential contributor is the long τ values estimated, as *T*_L_ is expected to vary less when τ is long (Ball, Cowan, & Farquhar, 1988). The long τ for *Guapira* leaves were a result of the combined low *g*_s_ and low LDMC (Figure 7, Figure S9); because water has a higher specific heat capacity than dry leaf matter, the higher water content of *Guapira* leaves causes a longer τ (Vogel, 2009).

### 4.3 Towards a better understanding of tropical leaf temperature behaviour

The link between functional traits and leaf thermoregulation has been highlighted in recent work (Michaletz et al., 2015, 2016). Here, we provide field‐based evidence for this link in the most detailed study of leaf energy balance in tropical montane forests to date and include variation in water use as a key component. The traits that we find important (leaf width, *g*_s_ at high *D*, and LDMC) may possibly connect other axes of plant functional variation (Reich, 2014)—the leaf economics spectrum (Wright et al., 2004) and plant hydraulics. Species that are able to maintain transpiration under high thermal stress conditions (high *T*_*A*_, PAR, and *D*) will require water to supply the transpiration stream from an efficient hydraulic system or from high water capacitance to avoid hydraulic failure. Avoiding extremes of *T*_L_ and maintaining open stomata will then have the benefit of keeping *T*_L_ closer to the temperature optima of photosynthesis, maintaining a CO_2_ supply, and all this while PAR is high to drive a high photosynthetic rate (Ball et al., 1988). Conversely, lower transpiration under high thermal stress conditions will prevent excessive water loss and therefore avoid risk of hydraulic failure through xylem embolism but increase risk of the leaf reaching a damaging high temperature threshold. Critical thresholds of photosynthetic activity vary by species (O'Sullivan et al., 2017). A recent study of critical thresholds of 41 co‐occurring tropical species found that variation was related to the leaf economics spectrum (Wright et al., 2004), with high LMA species showing higher temperature tolerance (Sastry & Barua, 2017). *Miconia* has significantly higher LMA than *Alchornea* (Figure 6), and it would be parsimonious if it also displays a higher critical temperature for damage to photosynthetic machinery. In summary, we hypothesize that trees at the *slow* end of the life‐history spectrum (Reich, 2014) are likely to reach higher leaf temperatures, have lower *g*_s_ and photosynthesis under high thermal stress conditions, have lower risk of hydraulic failure, and have a higher threshold for thermal damage, with the converse true of *fast* species.

If we are to understand the implications of climate change for tropical forests, it will be crucial to understand mechanisms of leaf thermoregulation and how this varies between species. We have based our findings on only a small, if detailed, dataset. There are very few comparable datasets available for tropical forests. More datasets exploring the full energy balance of tropical leaves from multiple sites with varying climatologies, and ideally over extended time periods, would certainly aid this. Beyond understanding current patterns of leaf temperatures, it is also necessary to understand the response of energy balance parameters to high *T*_A_ and CO_2_. For example, herbarium data for an Australian shrub species showed a reduction in leaf width over the last century (Guerin, Wen, & Lowe, 2012), which could mitigate increases in *T*_L_ due to increased *T*_A_. Conversely, declines in *g*_s_ are a common response of tree species to increased CO_2_, which, although potentially reducing water use, could lead to higher *T*_L_ (e.g., Barker et al., 2005; Warren et al., 2011). However, the extent of reductions in *g*_s_ under elevated CO_2_ varies with species (Way et al., 2015). In a study of seedlings of 10 tropical species, Cernusak et al. (2011) found reductions in *g*_s_ in all species in response to elevated CO_2_, but the reductions were larger for species with high *g*_s_ in ambient conditions. Warming may also cause changes in *g*_s_; results from warming experiments show a variety of responses—increases, decreases, and no change (Way et al., 2015)—and a recent meta‐analysis found decreases in stomatal density with higher *T*_A_ in trees but not in herbs (Yan, Zhong, & Shangguan, 2017). If trees do indeed decrease *g*_s_ under higher growth temperatures, this could result in further leaf warming beyond *T*_A_ increases but only if transpiration declines as well as *g*_s_, which is not certain given the expected rise in *D* with increased *T*_A_. Our understanding of the effects of combined CO_2_ and warming is even more limited. If both cause a decline in *g*_s_ separately, would the combined effect be additive leading to even greater reductions? The limited experimental data do not paint a clear picture (Way et al., 2015). A final question is whether leaves that reach higher temperatures are better adapted to cope with high temperatures and, therefore, increasing *T*_L_ would be less consequential than for low‐temperature species, or does the fact that leaf temperatures are already high mean that high‐temperature species are more at risk?

## 5 CONCLUSIONS

In this study, we made detailed measurements of leaf energy balance for three tree species in the montane Atlantic forest, Brazil. Our results show surprising high leaf‐to‐air temperature differences given the relatively low air temperatures, which we attribute to the high light conditions during the study. The higher radiation levels occurring at high elevations may contribute to the risks of climate change to tropical montane forests. We find differences in leaf thermoregulation between leaves of *Alchornea* and *Miconia*, which is attributable to lower transpiration under high thermal stress conditions for *Miconia* due to its wider leaves and stronger reduction of *g*_s_ with increasing *D*. Leaf energy balance modelling can be a powerful tool to understand variation between species in leaf thermoregulation, which will be necessary to model the impact of climate change on leaf physiology.

## Supporting information


**Appendix S1.** Leaf energy balance methods.
**Appendix S2.** Estimation of leaf boundary layer resistance from measured ΔT and rearrangement of the leaf energy balance equation.
**Table S1.** Details of sample leaves including leaf traits
**Figure S1.** Trend in temperature across the tropical forest biome. Statistically significant trends are shown with stippling. Data from CRU TS 3.24 (Harris et al., 2013). Triangle indicates field site
**Figure S2**. Photographs of samples leaves. a) thermocouple attachment to sample leaf *Alchornea*‐3, b) sample leaves *Alchornea*‐5 and *Alchornea*‐6, c) sample leaf *Miconia*‐3, d) cables from tower to leaves, PAR and *TA* sensors can be seen adjacent to tower.
**Figure S3.** Leaf boundary layer resistance to water (rbW) as a function of wind speed. Mean and SD of rbW in 0.1 m s‐1 bins are shown, excluding outliers and values where PAR < 200 μmol m‐2 s‐1. *Alchornea* one param a = 0.0171 ± 0.00005 SE, two param a = 0.0307 ± 0.0003 SE, b = 0.210 ± 0.004 SE. *Miconia* one param a = 0.0215 ± 0.00008 SE, two param a = 0.0327 ± 0.0002 SE, b = 0.069 ± 0.005 SE. See Appendix 2 for further details.
**Figure S4.** PAR as measured with a hand‐held sensor at the leaf surface against PAR as recorded from the nearest sensor on the tower.
**Figure S5.** Validation of thermocouple data against leaf temperature as measured on the abaxial surface with IR thermometer. Each panel is a different leaf (labeled in top left corner). Grey dash line – y = x. Solid coloured line ‐ regression line forced through zero. Slope (a) ± 95% CI is shown. Slope is significantly different from zero for leaves A3, G2 and G3. Two leaves (M5 and M6) are not shown due to lack of IR thermometer data as a consequence of petiole breakage during the study.
**Figure S6.** Microclimate variables through the vertical profile during the field monitoring period. Colours denote measurement heights. PAR and *TA* where collected at 10 second intervals. RH was collected at 1 minute intervals. Due to a sensor malfunction, RH data was not available until 5 October; data prior to 5 October was estimated based on the strong relationship between RH and *TA* in the available data.
**Figure S7.** Histograms of daytime TL for each leaf. Title for each panel shows the leaf ID. Max and mean daytime TL are shown.
**Figure S8**. Histograms of daytime *ΔT* for each leaf. Title for each panel shows the leaf ID. Max and mean daytime *ΔT* are shown. %high indicates the percentage of *ΔT* measurements above 2°C, %low indicates the percentage of *ΔT* measurements below −2°C.
**Figure S10**. Relationships between air temperature and observed *ΔT* (a, b) and estimated *ΔTe* (c, d) for *Alchornea* (a, c) and *Miconia* (b, d). Solid line – modelled relationship for the plotted species, dash line – modelled relationship for the alternative species. Statistical models are linear mixed effects model with leaf as a random factor. R2 is the marginal pseudo R2.
**Figure S11**. Relationships between VPD and observed *ΔT* (a, b) and estimated *ΔTe* (c, d) for *Alchornea* (a, c) and *Miconia* (b, d). Solid line – modelled relationship for the plotted species, dash line – modelled relationship for the alternative species. Statistical models are linear mixed effects model with leaf as a random factor. R2 is the marginal pseudo R2.
**Figure S12**. Relationships between wind speed and observed *ΔT* (a, b) and estimated *ΔTe* (c, d) for *Alchornea* (a, c) and *Miconia* (b, d). Solid line – modelled relationship for the plotted species, dash line – modelled relationship for the alternative species. Statistical models are linear mixed effects model with leaf as a random factor. R2 is the marginal pseudo R2.
**Figure S13**. Relationships between PAR and observed *TL* (a, b) and estimated *TL* (c, d) for *Alchornea* (a, c) and *Miconia* (b, d). Solid line – modelled relationship for the plotted species, dash line – modelled relationship for the alternative species. Statistical models are linear mixed effects model with leaf as a random factor. R2 is the marginal pseudo R2.
**Figure S14**. Relationships between *TA* and observed *TL* (a, b) and estimated *TL* (c, d) for *Alchornea* (a, c) and *Miconia* (b, d). Solid line – modelled relationship for the plotted species, dash line – modelled relationship for the alternative species. Statistical models are linear mixed effects model with leaf as a random factor. R2 is the marginal pseudo R2.
**Figure S15**. Relationships between VPD and observed *TL* (a, b) and estimated *TL* (c, d) for *Alchornea* (a, c) and *Miconia* (b, d). Solid line – modelled relationship for the plotted species, dash line – modelled relationship for the alternative species. Statistical models are linear mixed effects model with leaf as a random factor. R2 is the marginal pseudo R^2^.
**Figure S16**. Relationships between wind speed and observed *TL* (a, b) and estimated *TL* (c, d) for *Alchornea* (a, c) and *Miconia* (b, d). Solid line – modelled relationship for the plotted species, dash line – modelled relationship for the alternative species. Statistical models are linear mixed effects model with leaf as a random factor. R2 is the marginal pseudo R2.
**Figure S17.** Relationship between PAR and *ΔTe* of *Alchornea* and *Miconia* using the species‐specific parameterisations (coral and light blue lines respectively), and *ΔTe* of *Alchornea* using different aspects of the *Miconia* parameterisation.
**Figure S18**. Comparison of the maximum PAR measured in the profile used in this study and an independent dataset from a weather station mounted at 27 m on the same tower using a Li‐Cor LI‐190R quantum sensor. The weather station provided 10 minute average PAR values, and hence the profile values were recalculated as 10 minute averages for this comparison. The left‐hand panel shows the time series during the measurement period, red – PAR from weather station, black – maximum PAR recorded in the profile. The right‐hand panel shows the close relationship between the two datasets, with possibly some underestimation in the profile as the highest sensor (25 m) was below the weather station (27 m).
