# Supplementary material for: Differences in leaf thermoregulation and water use strategies between three co‐occurring Atlantic forest tree species
Source: Plant Cell Environ. 2018 May 3;41(7):1618–31. doi: 10.1111/pce.13208 (PMC6032932; doi:10.1111/pce.13208)
Supplement: Supplementary file 1 — Appendix S1. Leaf energy balance methods. Appendix S2. Estimation of leaf boundary layer resistance from measured ΔT and rearrangement of the leaf energy balance equation. Table S1. Details of sample leaves including leaf traits Figure S1. Trend in temperature across the tropical forest biome. Statistically significant trends are shown with stippling. Data from CRU TS 3.24 (Harris et al., 2013). Triangle indicates field site Figure S2. Photographs of samples leaves. a) thermocouple attachment to sample leaf Alchornea‐3, b) sample leaves Alchornea‐5 and Alchornea‐6, c) sample leaf Miconia‐3, d) cables from tower to leaves, PAR and TA sensors can be seen adjacent to tower. Figure S3. Leaf boundary layer resistance to water (rbW) as a function of wind speed. Mean and SD of rbW in 0.1 m s‐1 bins are shown, excluding outliers and values where PAR < 200 μmol m‐2 s‐1. Alchornea one param a = 0.0171 ± 0.00005 SE, two param a = 0.0307 ± 0.0003 SE, b = 0.210 ± 0.004 SE. Miconia one param a = 0.0215 ± 0.00008 SE, two param a = 0.0327 ± 0.0002 SE, b = 0.069 ± 0.005 SE. See Appendix 2 for further details. Figure S4. PAR as measured with a hand‐held sensor at the leaf surface against PAR as recorded from the nearest sensor on the tower. Figure S5. Validation of thermocouple data against leaf temperature as measured on the abaxial surface with IR thermometer. Each panel is a different leaf (labeled in top left corner). Grey dash line – y = x. Solid coloured line ‐ regression line forced through zero. Slope (a) ± 95% CI is shown. Slope is significantly different from zero for leaves A3, G2 and G3. Two leaves (M5 and M6) are not shown due to lack of IR thermometer data as a consequence of petiole breakage during the study. Figure S6. Microclimate variables through the vertical profile during the field monitoring period. Colours denote measurement heights. PAR and TA where collected at 10 second intervals. RH was collected at 1 minute intervals. Due to a sensor malfunction, RH data w [file PCE-41-1618-s001.pdf]

**Supporting Information for 'Differences in leaf thermoregulation and water-use strategies between three co-occurring Atlantic forest tree species'**
**Fauset *et al.***

**Contains**
Table S1
Figures S1-S18
Appendix 1 – Leaf energy balance methods.
Appendix 2 - Estimation of leaf boundary layer resistance from measured $\Delta T$ and rearrangement of the leaf energy balance equation.

**Table S1.** Details of sample leaves including leaf traits.

| Leaf ID | Species | No. Sampling Days | Height (m) | Angle (° from horizontal)* | Orientation (°) | Width (cm) | Area ($cm^2$) | LMA ($g\ m^{-2}$) | LDMC ($g\ g^{-1}$) | Mean Daytime PAR ($\mu mol\ m^{-2}\ s^{-1}$)** |
|---|---|---|---|---|---|---|---|---|---|---|
| A1 | *Alchornea* | 8 | 18.2 | 24 | 170 | 9.0 | 71.0 | 93.0 | 0.38 | 554 |
| A2 | *Alchornea* | 8 | 18.2 | 5 | 77 | 7.9 | 57.8 | 89.9 | 0.37 | 554 |
| A3 | *Alchornea* | 8 | 17.7 | 43 | 170 | 6.5 | 40.4 | 108.8 | 0.39 | 480 |
| A4 | *Alchornea* | 10 | 17.7 | 42 | 195 | 6.3 | 37.1 | 80.9 | 0.37 | 480 |
| A5 | *Alchornea* | 8 | 18.2 | 27 | 226 | 6.5 | 68.3 | 51.3 | 0.35 | 554 |
| A6 | *Alchornea* | 8 | 18.2 | 5 | 300 | 5.1 | 29.7 | 84.2 | 0.37 | 554 |
| A7 | *Alchornea* | 10 | 15.25 | 40 | 197 | 6.3 | 37.5 | 88.0 | 0.38 | 348 |
| A8 | *Alchornea* | 10 | 15.25 | 65 | 150 | 6.2 | 38.2 | 83.9 | 0.37 | 348 |
| A9 | *Alchornea* | 10 | 9.7 | 55 | 234 | 7.1 | 55.2 | 61.6 | 0.34 | 205 |
| A10 | *Alchornea* | 10 | 9.7 | 23 | 293 | 5.5 | 31.6 | 66.4 | 0.35 | 205 |
| M1 | *Miconia* | 10 | 15.0 | 36 | 94 | 13 | 230.3 | 162.4 | 0.40 | 348 |
| M2 | *Miconia* | 10 | 15.0 | 39 | 338 | 15.3 | 275.7 | 163.9 | 0.41 | 348 |
| M3 | *Miconia* | 8 | 15.2 | 30 | 90 | 13.2 | 188.3 | 176.8 | 0.43 | 348 |
| M5 | *Miconia* | 4 | 15.2 | 50 | 25 | 19.2 | 400.0 | 204.7 | 0.44 | 348 |
| M6 | *Miconia* | 6 | 15.2 | 50 | 330 | 17.5 | 318.9 | 196.0 | 0.43 | 348 |
| M7 | *Miconia* | 10 | 14.7 | 28 | 311 | 13.4 | 195.6 | 170.2 | 0.42 | 257 |
| M8 | *Miconia* | 10 | 14.7 | 34 | 31 | 11.2 | 186.3 | 171.7 | 0.42 | 257 |
| M9 | *Miconia* | 10 | 9.6 | 7 | 11 | 18.7 | 409.8 | 167.4 | 0.44 | 205 |
| M10 | *Miconia* | 10 | 9.4 | 40 | 60 | 22.7 | 517.6 | 168.3 | 0.42 | 205 |
| G1 | *Guapira* | 10 | 2.9 | 90 | 240 | 4.2 | 26.8 | 85.9 | 0.20 | 77 |
| G2 | *Guapira* | 10 | 2.9 | 52 | 240 | 4.7 | 25.2 | 63.5 | 0.16 | 77 |
| G3 | *Guapira* | 10 | 2.9 | 86 | 240 | 3.3 | 20.7 | 72.4 | 0.19 | 77 |
| G4 | *Guapira* | 8 | 2.9 | 90 | 240 | 4.4 | 30.3 | 89.3 | 0.20 | 77 |

* Angle given is the mean of a minimum of 5 measurements.

** Based on PAR measurements at the nearest sensor.

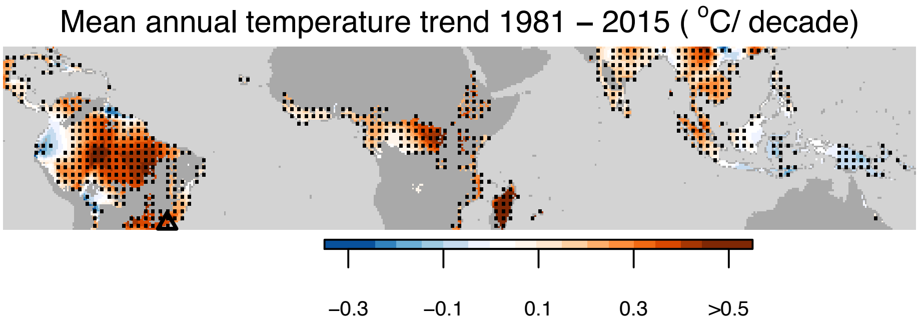


**Figure S1.** Trend in temperature across the tropical forest biome. Statistically significant trends are shown with stippling. Data from CRU TS 3.24 (Harris *et al*., 2013). Triangle indicates field site.

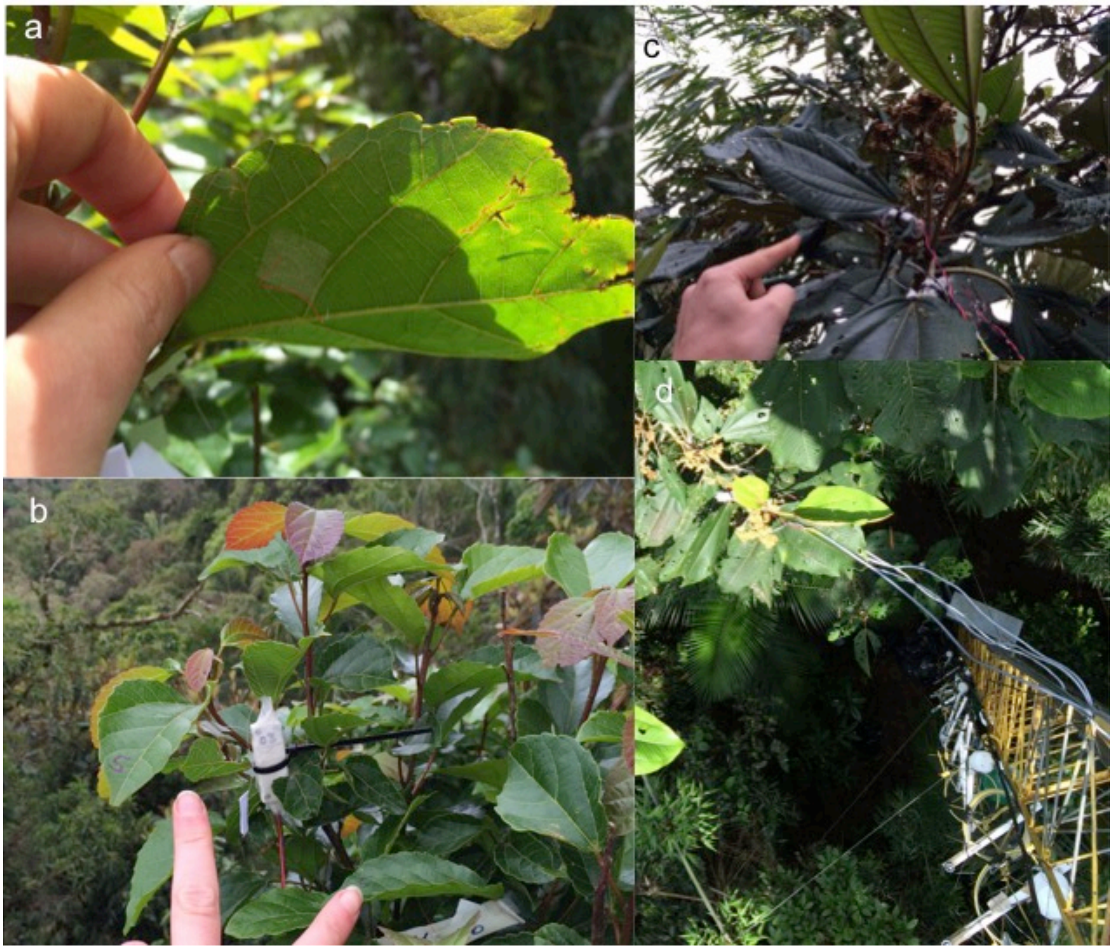

**Figure S2**. Photographs of samples leaves. a) thermocouple attachment to sample leaf *Alchornea*-3, b) sample leaves *Alchornea*-5 and *Alchornea*-6, c) sample leaf *Miconia*-3, d) cables from tower to leaves, PAR and $T_A$ sensors can be seen adjacent to tower.

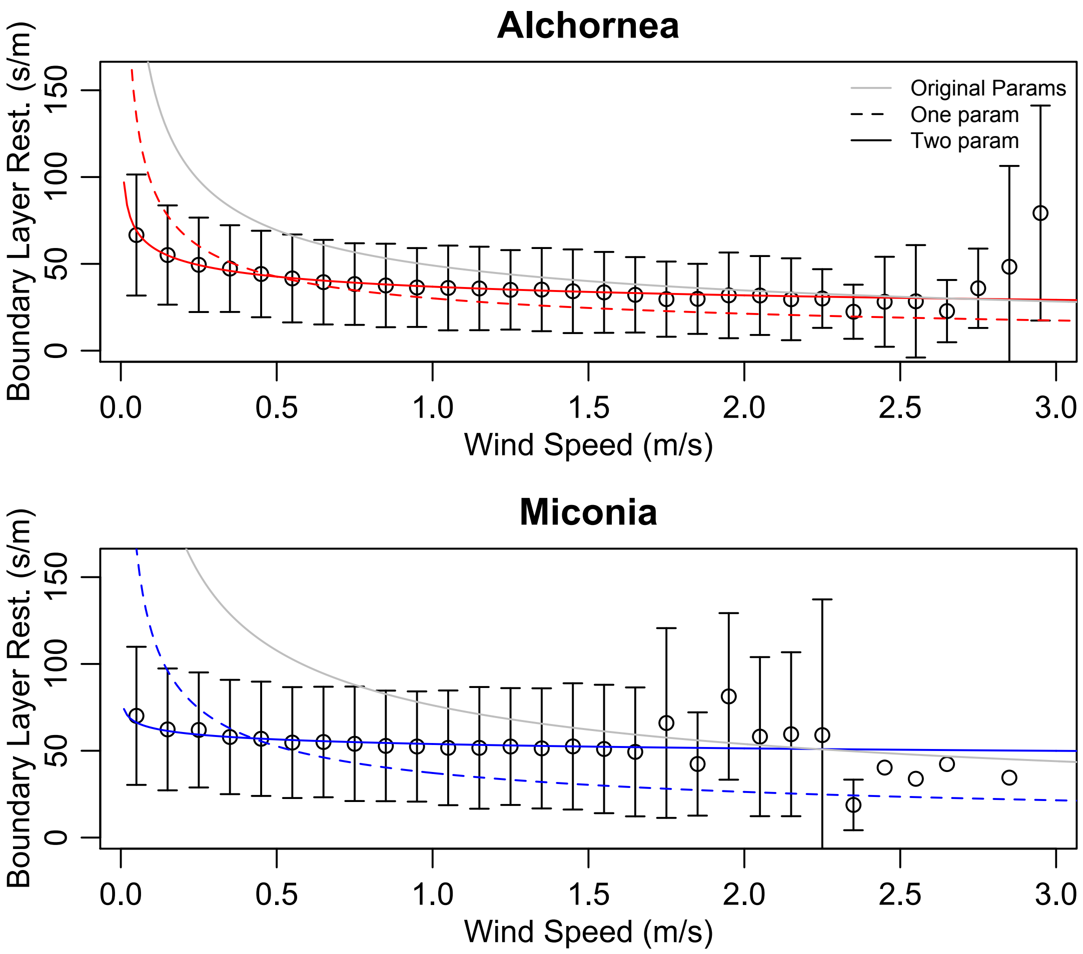


**Figure S3.** Leaf boundary layer resistance to water ($r_{bW}$) as a function of wind speed. Mean and SD of $r_{bW}$ in 0.1 m $s^{-1}$ bins are shown, excluding outliers and values where PAR < 200 µmol $m^{-2}$ $s^{-1}$. *Alchornea* one param a = 0.0171 ± 0.00005 SE, two param a = 0.0307 ± 0.0003 SE, b = 0.210 ± 0.004 SE. *Miconia* one param a = 0.0215 ± 0.00008 SE, two param a = 0.0327 ± 0.0002 SE, b = 0.069 ± 0.005 SE. See Appendix 2 for further details.

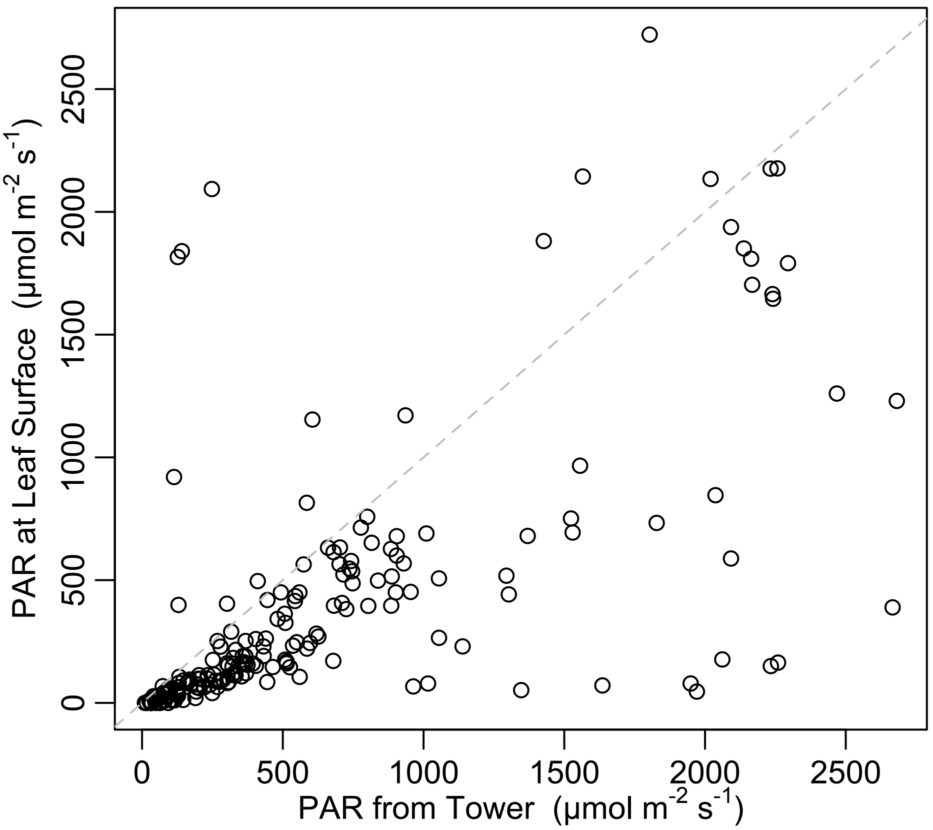


**Figure S4.** PAR as measured with a hand-held sensor at the leaf surface against PAR as recorded from the nearest sensor on the tower.

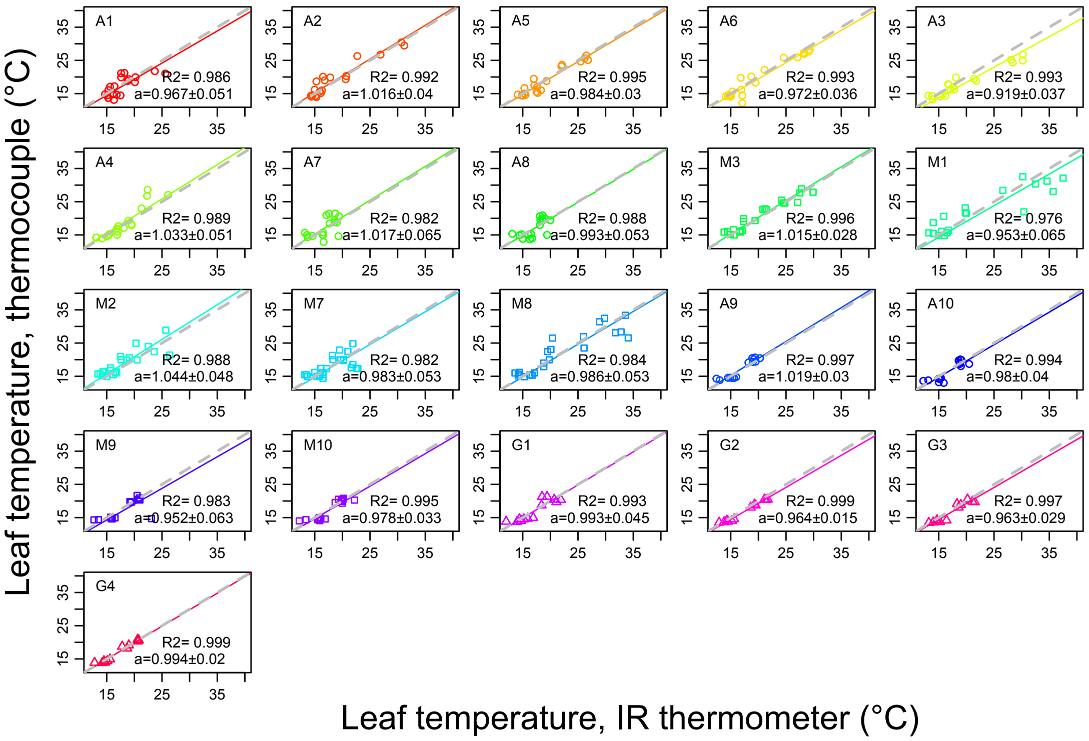


**Figure S5.** Validation of thermocouple data against leaf temperature as measured on the abaxial surface with IR thermometer. Each panel is a different leaf (labeled in top left corner). Grey dash line – y=x. Solid coloured line - regression line forced through zero. Slope (a) ± 95 % CI is shown. Slope is significantly different from zero for leaves A3, G2 and G3. Two leaves (M5 and M6) are not shown due to lack of IR thermometer data as a consequence of petiole breakage during the study.

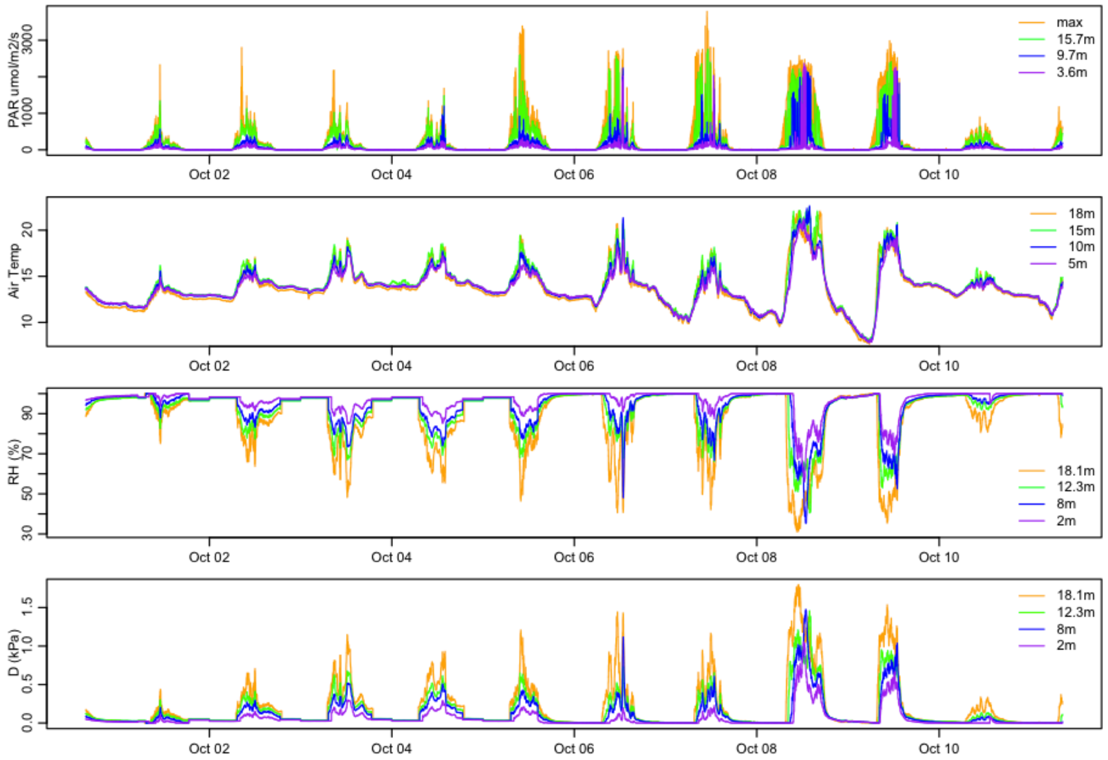


**Figure S6.** Microclimate variables through the vertical profile during the field monitoring period. Colours denote measurement heights. PAR and $T_A$ where collected at 10 second intervals. RH was collected at 1 minute intervals. Due to a sensor malfunction, RH data was not available until 5 October; data prior to 5 October was estimated based on the strong relationship between RH and $T_A$ in the available data.

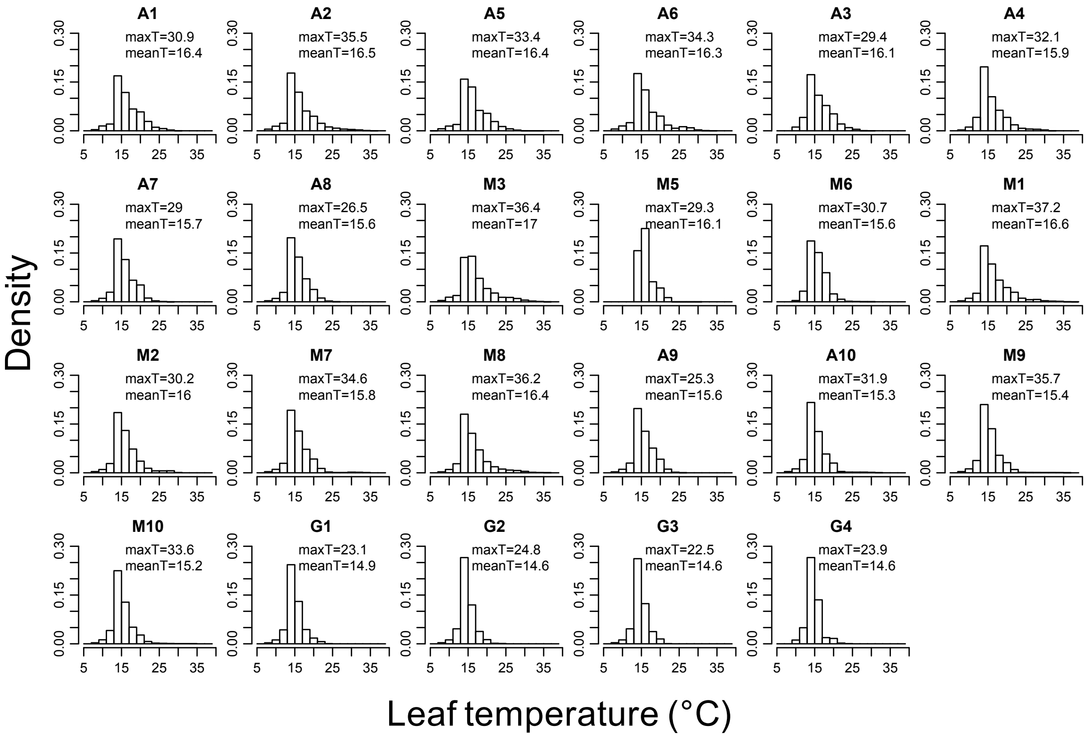


**Figure S7.** Histograms of daytime $T_L$ for each leaf. Title for each panel shows the leaf ID. Max and mean daytime $T_L$ are shown.

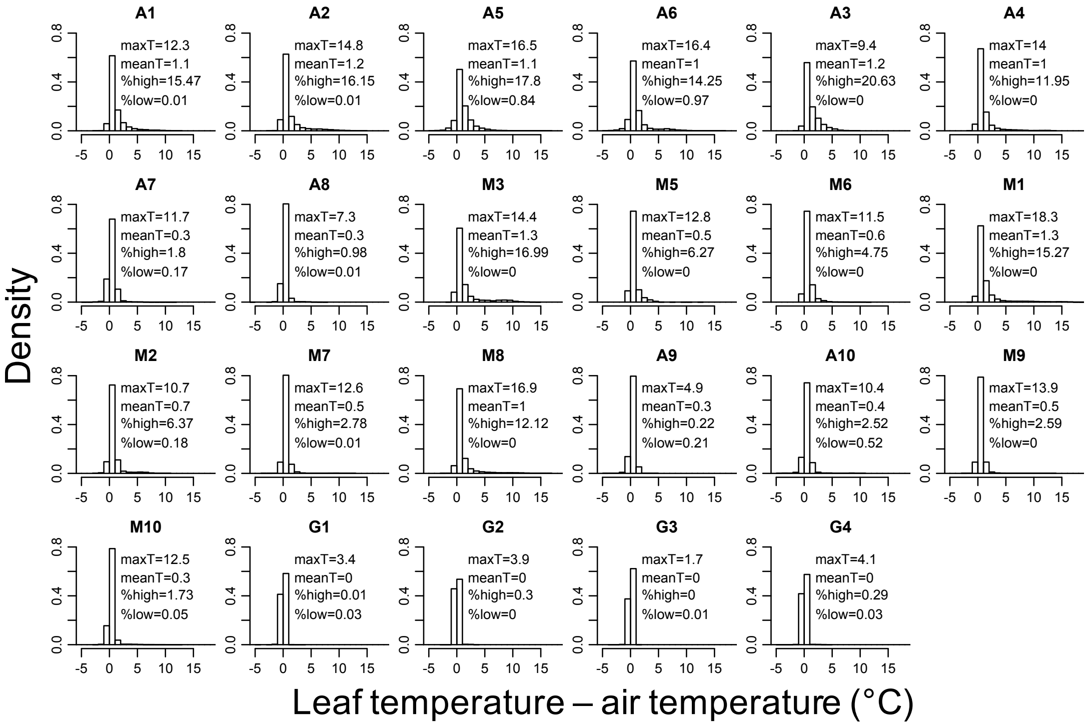


**Figure S8**. Histograms of daytime $\Delta T$ for each leaf. Title for each panel shows the leaf ID. Max and mean daytime $\Delta T$ are shown. %high indicates the percentage of $\Delta T$ measurements above 2 °C, %low indicates the percentage of $\Delta T$ measurements below -2 °C.

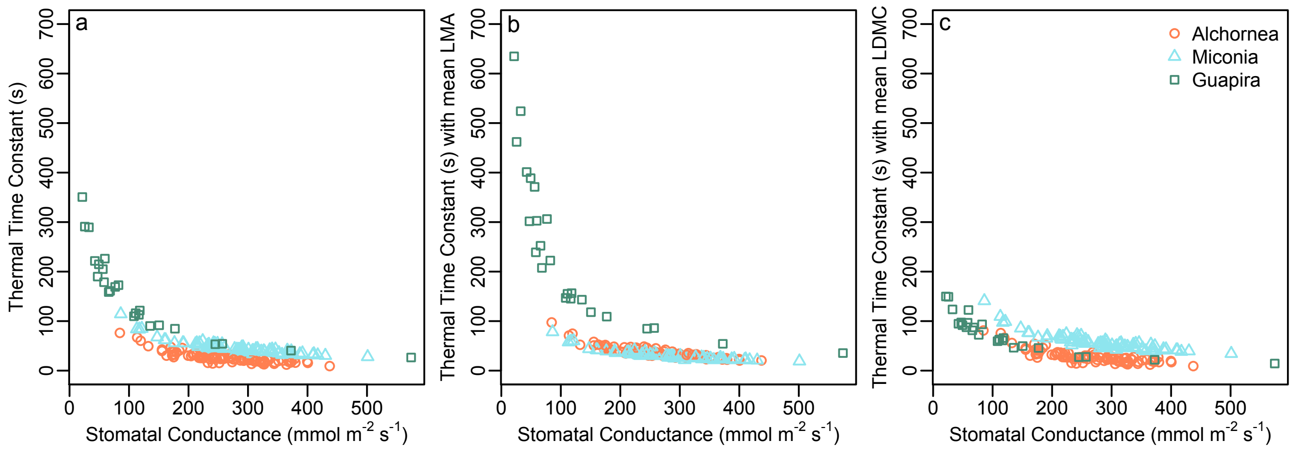


**Figure S9**. Thermal time constant (τ) estimated using a) the observed parameters, b) the mean leaf mass per area (LMA) applied to all species, and c) the mean leaf dry matter content (LDMC) applied to all species.

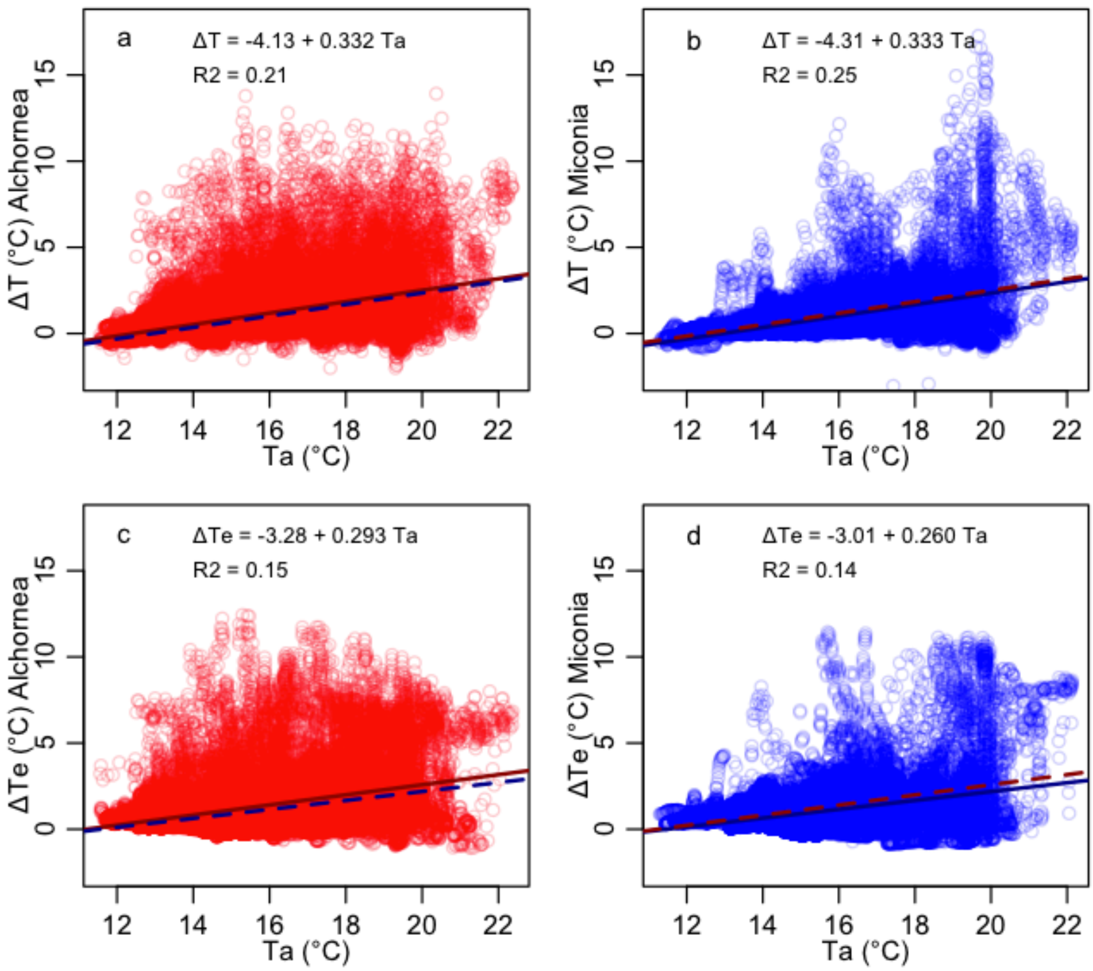


**Figure S10**. Relationships between air temperature and observed $\Delta T$ (a, b) and estimated $\Delta T_e$ (c, d) for *Alchornea* (a, c) and *Miconia* (b, d). Solid line – modelled relationship for the plotted species, dash line – modelled relationship for the alternative species. Statistical models are linear mixed effects model with leaf as a random factor. $R^2$ is the marginal pseudo $R^2$.

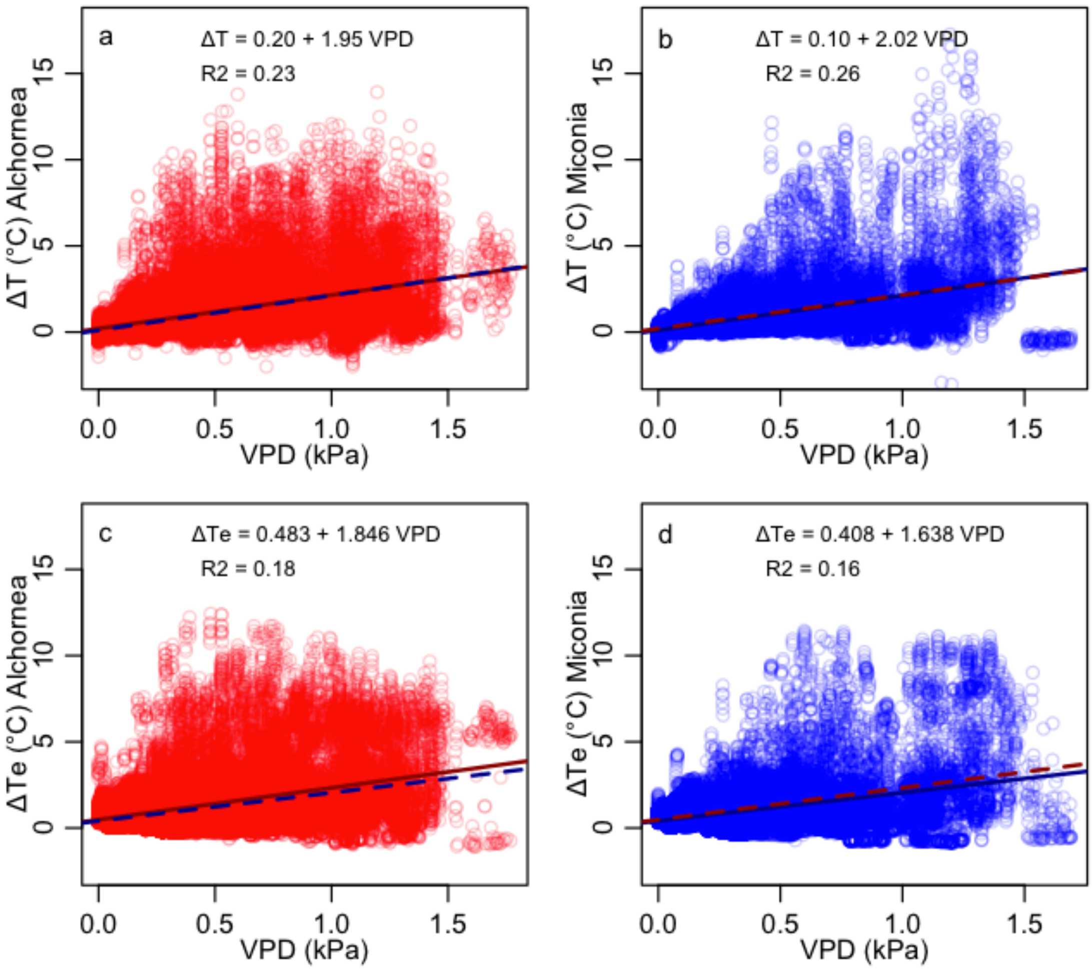


**Figure S11**. Relationships between VPD and observed $\Delta T$ (a, b) and estimated $\Delta T_e$ (c, d) for *Alchornea* (a, c) and *Miconia* (b, d). Solid line – modelled relationship for the plotted species, dash line – modelled relationship for the alternative species. Statistical models are linear mixed effects model with leaf as a random factor. $R^2$ is the marginal pseudo $R^2$.

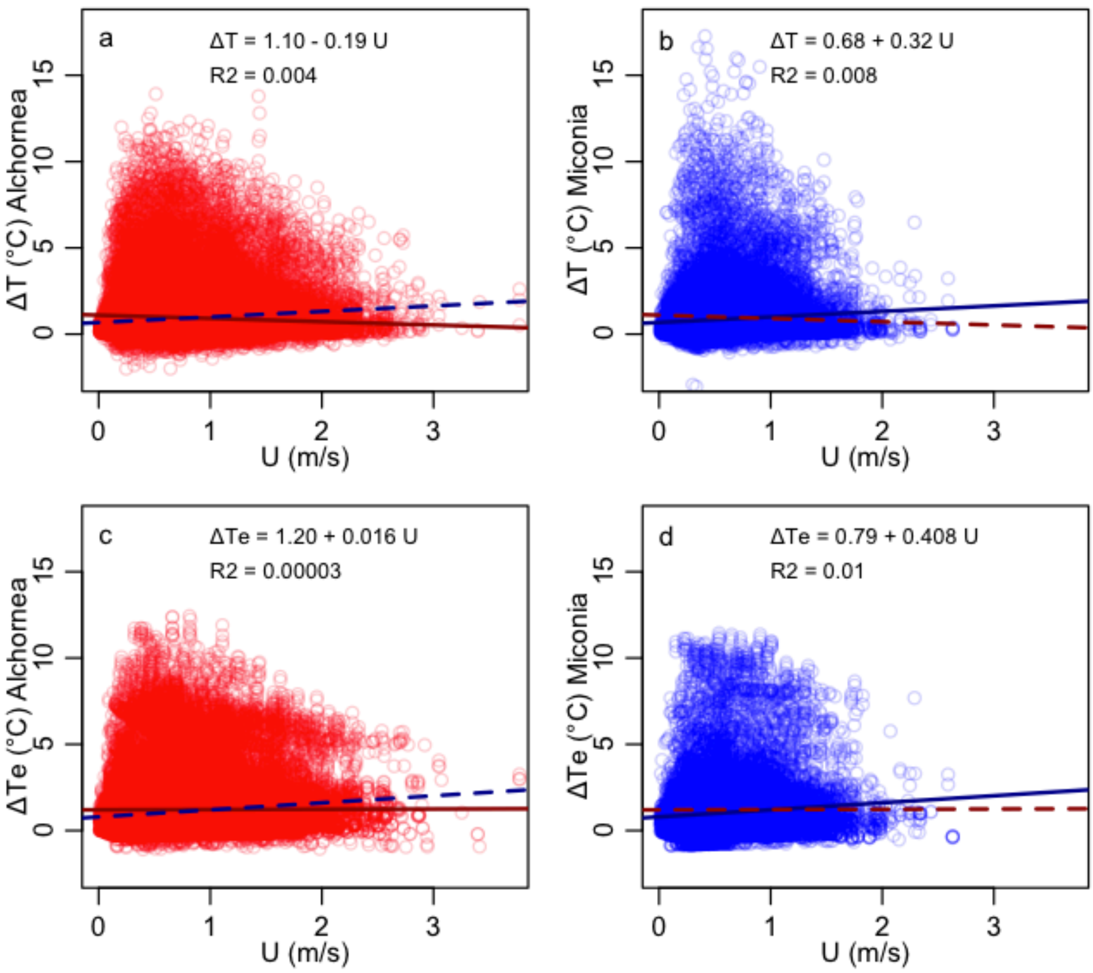


**Figure S12**. Relationships between wind speed and observed $\Delta T$ (a, b) and estimated $\Delta T_e$ (c, d) for *Alchornea* (a, c) and *Miconia* (b, d). Solid line – modelled relationship for the plotted species, dash line – modelled relationship for the alternative species. Statistical models are linear mixed effects model with leaf as a random factor. $R^2$ is the marginal pseudo $R^2$.

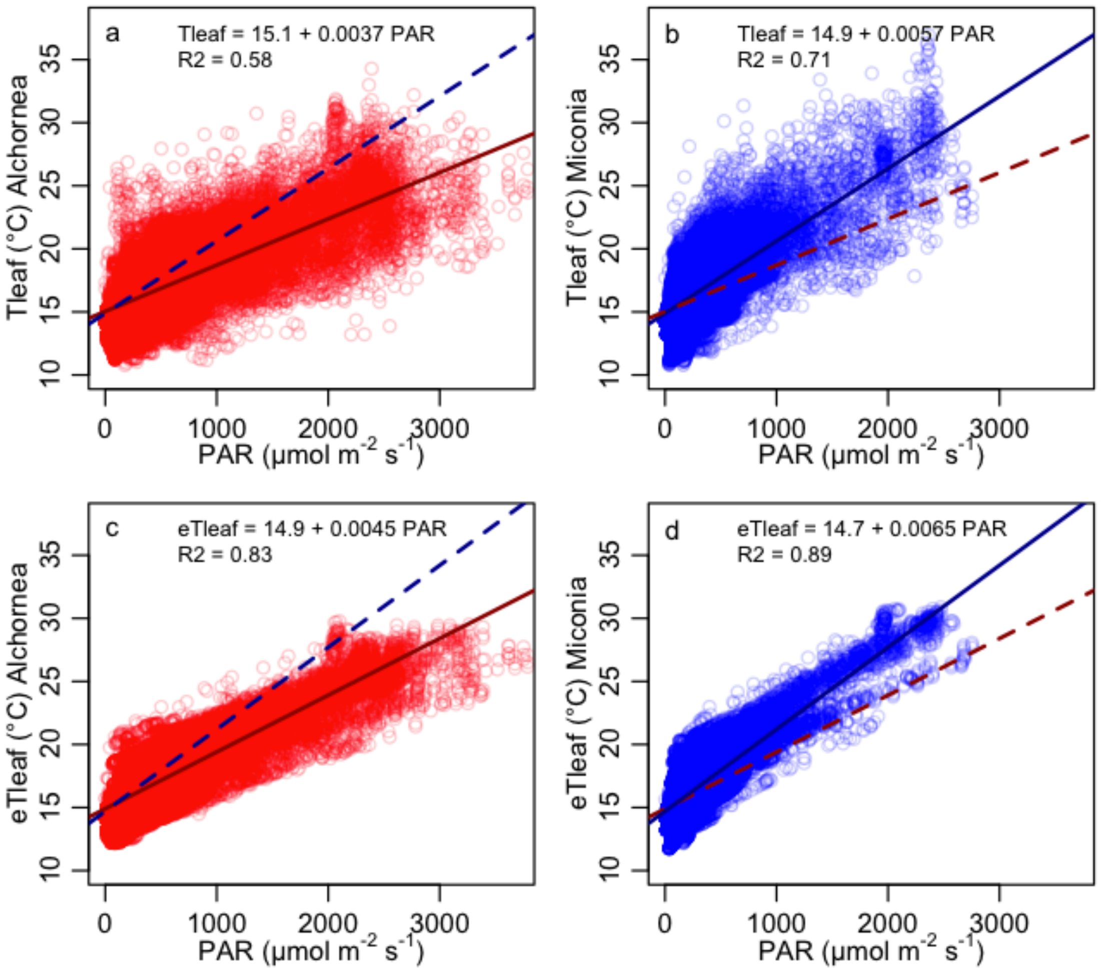


**Figure S13**. Relationships between PAR and observed $T_L$ (a, b) and estimated $T_L$ (c, d) for *Alchornea* (a, c) and *Miconia* (b, d). Solid line – modelled relationship for the plotted species, dash line – modelled relationship for the alternative species. Statistical models are linear mixed effects model with leaf as a random factor. $R^2$ is the marginal pseudo $R^2$.

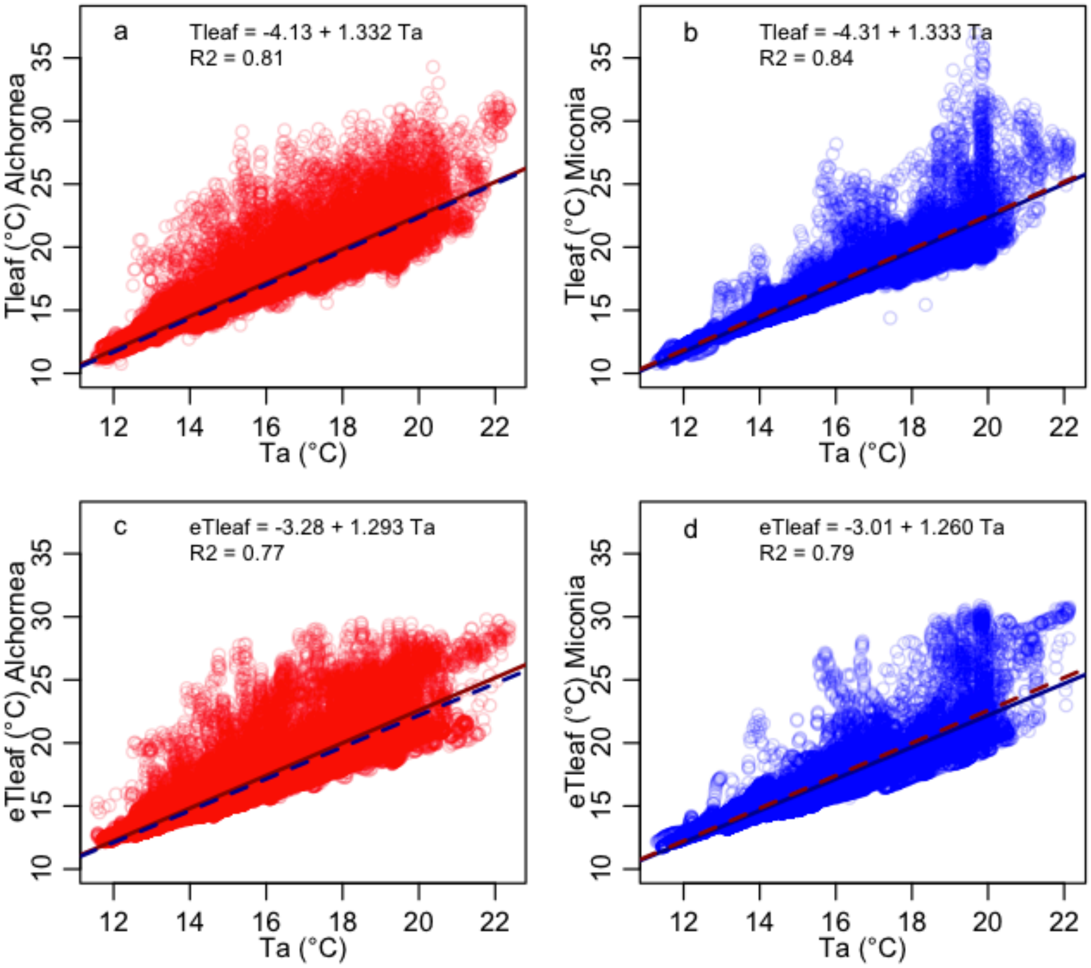


**Figure S14**. Relationships between $T_A$ and observed $T_L$ (a, b) and estimated $T_L$ (c, d) for *Alchornea* (a, c) and *Miconia* (b, d). Solid line – modelled relationship for the plotted species, dash line – modelled relationship for the alternative species. Statistical models are linear mixed effects model with leaf as a random factor. $R^2$ is the marginal pseudo $R^2$.

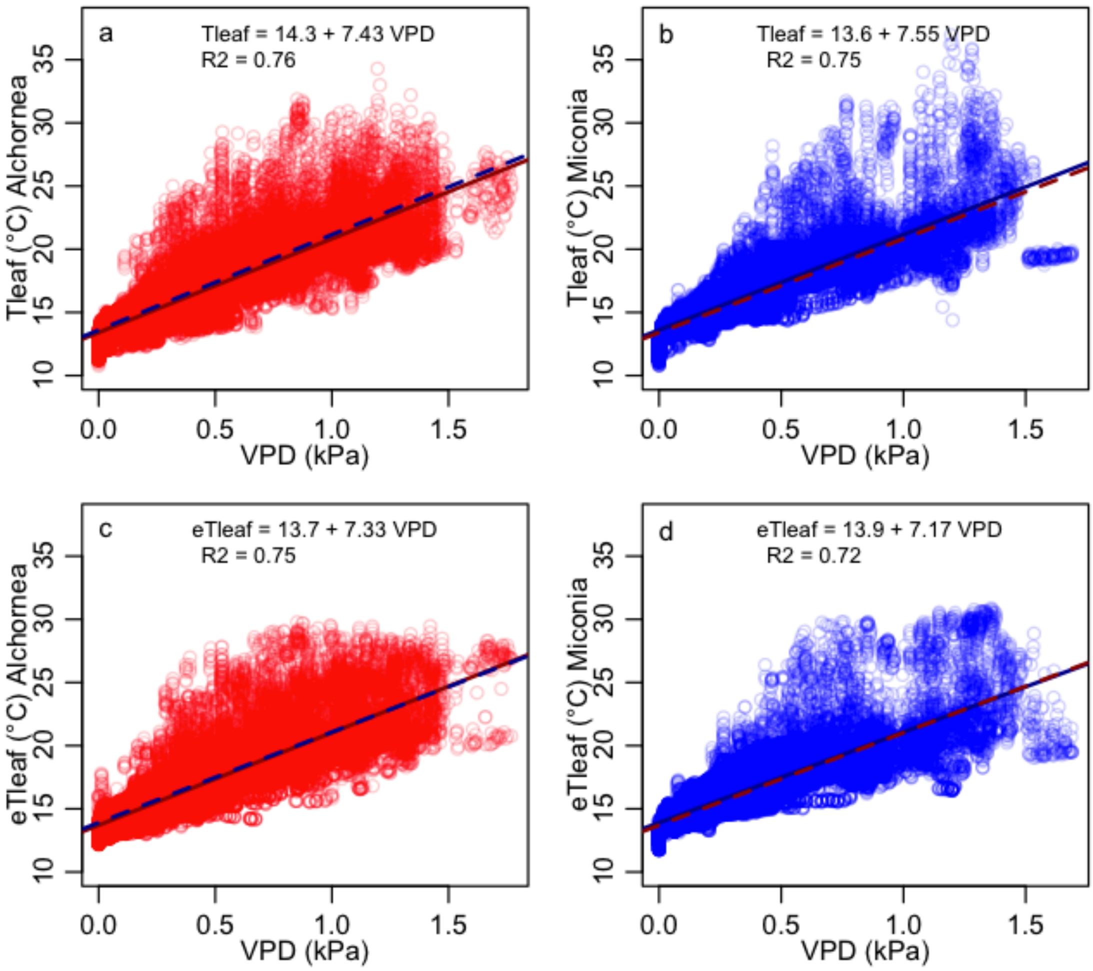


**Figure S15**. Relationships between VPD and observed $T_L$ (a, b) and estimated $T_L$ (c, d) for *Alchornea* (a, c) and *Miconia* (b, d). Solid line – modelled relationship for the plotted species, dash line – modelled relationship for the alternative species. Statistical models are linear mixed effects model with leaf as a random factor. $R^2$ is the marginal pseudo $R^2$.

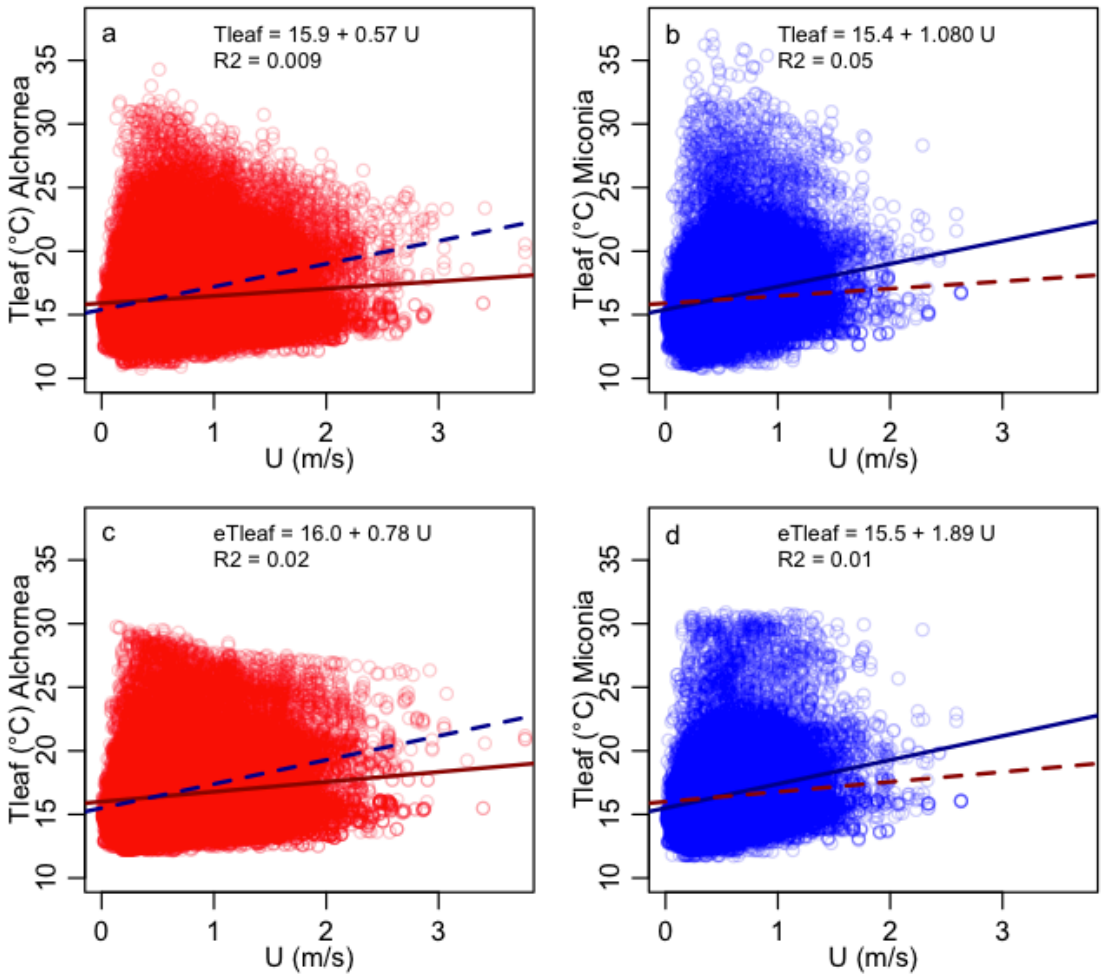


**Figure S16**. Relationships between wind speed and observed $T_L$ (a, b) and estimated $T_L$ (c, d) for *Alchornea* (a, c) and *Miconia* (b, d). Solid line – modelled relationship for the plotted species, dash line – modelled relationship for the alternative species. Statistical models are linear mixed effects model with leaf as a random factor. $R^2$ is the marginal pseudo $R^2$.

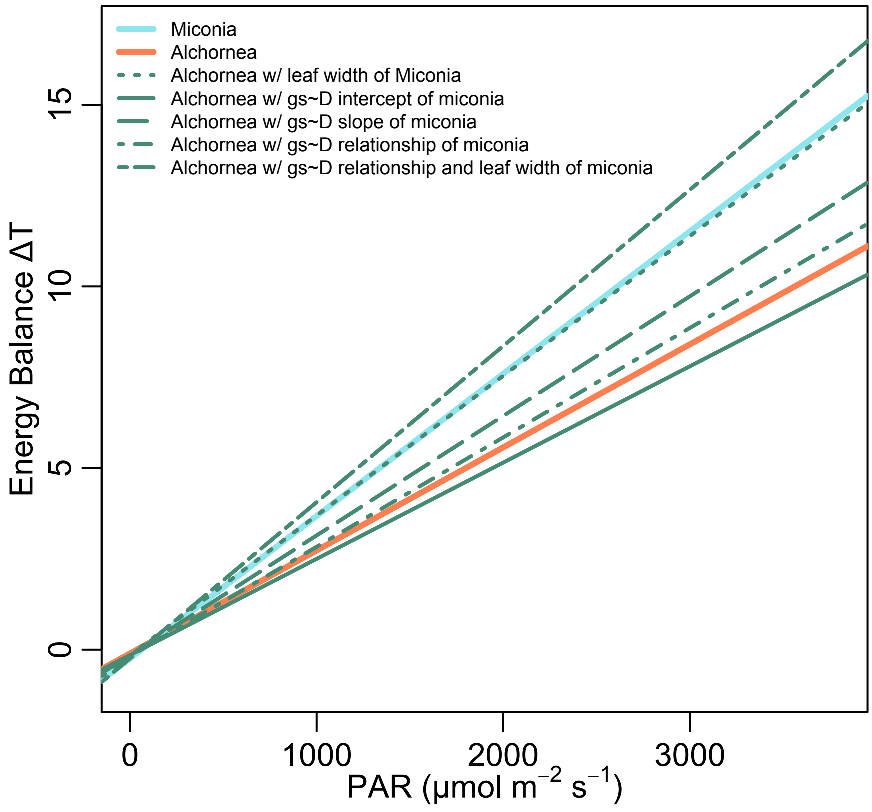


**Figure S17.** Relationship between PAR and $\Delta T_e$ of *Alchornea* and *Miconia* using the species-specific parameterisations (coral and light blue lines respectively), and $\Delta T_e$ of *Alchornea* using different aspects of the *Miconia* parameterisation.

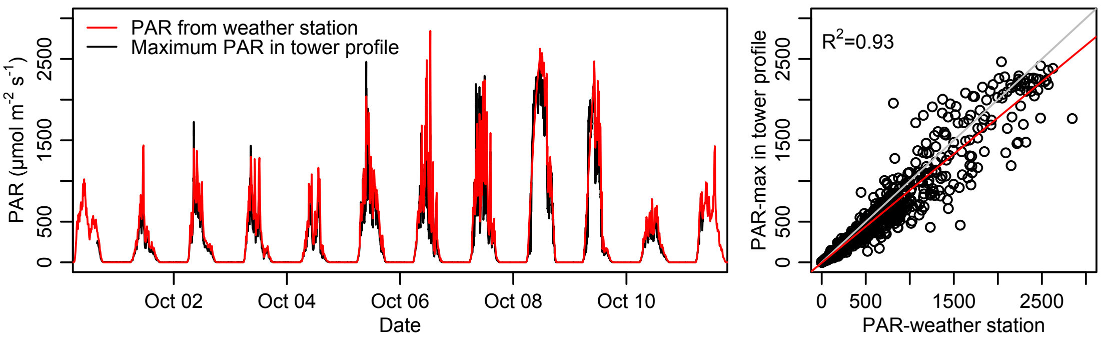


**Figure S18**. Comparison of the maximum PAR measured in the profile used in this study and an independent dataset from a weather station mounted at 27 m on the same tower using a Li-Cor LI-190R quantum sensor. The weather station provided 10 minute average PAR values, and hence the profile values were recalculated as 10 minute averages for this comparison. The left-hand panel shows the time series during the measurement period, red – PAR from weather station, black – maximum PAR recorded in the profile. The right-hand panel shows the close relationship between the two datasets, with possibly some underestimation in the profile as the highest sensor (25 m) was below the weather station (27 m).

## Appendix 1 – Leaf energy balance methods

Net isotropic radiation (assuming the leaf is at air temperature) energy gain ($R_{ni}$, W m$^{-2}$) is defined following Jones (1992) as

$$R_{ni} = R_S \cdot (1 + \rho_S) \cdot \alpha_S + \varepsilon_{sky} \sigma T_{sky}^4 - \varepsilon_{leaf} \sigma T_A^4 \quad \text{(S1)}$$

where $R_S$ is the incoming shortwave radiation (W m$^{-2}$) estimated from PAR using a conversion factor (Jones, 1992), $\alpha_S$ is the short wave absorption coefficient for the leaf (0.5), $\rho_S$ is the reflection coefficient of the forest (0.12), $\varepsilon_{sky}$ is the sky emissivity (assumed to be constant at 0.97), σ is the Stephan-Boltzman constant, $T_{sky}$ is the sky temperature and $\varepsilon_{leaf}$ is the emissivity of the leaf (assumed to be 0.95). For the estimation of incoming longwave radiation sky temperature was assumed equal to $T_A$ measured at the nearest $T_A$ sensor to the leaf. This may somewhat overestimate in the incoming longwave radiation as the sky may be cooler than the air near the leaf.

The boundary layer resistance to heat and radiation ($r_{b,HR}$) is defined as

$$r_{b,HR} = 1/(g_{b,H} + g_{b,R}) \quad \text{(S2)}$$

where $g_{b,H}$ is the boundary layer conductance to heat (m s$^{-1}$)

$$g_{b,H} = 0.0105 \left( {}^{U}/_{W} \right)^{0.5} \quad \text{(S3)}$$

where $U$ is wind speed (m s$^{-1}$) and $W$ is leaf width (m), and $g_{b,R}$ is the boundary layer conductance to radiation (m s$^{-1}$)

$$g_{b,R} = 4\varepsilon\sigma {T_A}^3 / \rho_a c_p \quad \text{(S4)}$$

$$r_{b,W} = 2/g_{b,H} \quad \text{(S5)}$$

$$r_{l,W} = 1/\left( {}^{g_s}/_{d} \right) \quad \text{(S6)}$$

where $g_s$ is stomatal conductance (mmol m$^{-2}$ s$^{-1}$) and $d$ is a unit conversion factor.

Some constants (λ, γ, $\rho_a$) are temperature sensitive and were evaluated from air temperature to the nearest 5°C using values from Jones (1992).

**Appendix 2 – Estimation of leaf boundary layer resistance from measured *ΔT* and rearrangement of the leaf energy balance equation.**

To estimate leaf boundary layer resistance, the energy balance equation (equation S7) was solved for $r_{bW}$

$$\Delta T = \frac{r_{bH}(r_{bW}+r_{lW})\gamma R_n}{\rho_a c_p[\gamma(r_{bW}+r_{lW})+s r_{b,HR}]} - \frac{r_{bH} D}{\gamma(r_{bW}+r_{lW})+s r_{bH}} \quad \text{(S7)}$$

Equation S7 differs slightly from equation 3 in the main text because we are including the measured *ΔT* and can calculate net radiation ($R_n$) from the observed leaf temperature, an unknown in equation 3.
$r_{bH}$, the boundary layer resistance to heat, is equal to half the value of $r_{bW}$ (accounting for the loss of heat from both sides of the leaf, but water only from one side). Therefore, we substitute $0.5 r_{bW}$ into equation S7 for $r_{bH}$ and solve for $r_{bW}$. This results in a quadratic expression (equation S8, below).

$$r_{bW_{1,2}} = \frac{-\left(r_{lW} - \frac{2\cdot\rho_a c_p\cdot\Delta T}{R_n} - \frac{\rho_a c_p\cdot D}{\gamma R_n} - \frac{\rho_a c_p\cdot\Delta T\cdot s}{\gamma R_n}\right) \pm \sqrt{\left(r_{lW} - \frac{2\cdot\rho_a c_p\cdot\Delta T}{R_n} - \frac{\rho_a c_p\cdot D}{\gamma R_n} - \frac{\rho_a c_p\cdot\Delta T\cdot s}{\gamma R_n}\right)^2 + 4\frac{2\rho_a c_p\cdot\Delta T\cdot r_{lW}}{R_n}}}{2}$$

Evaluation of equation S8 showed that only addition of the square root term produced viable values for $r_{bW}$ (subtraction resulted in negative resistances).

Examination of the values of $r_{bW}$ produced showed a number of outliers (unrealistically high or negative resistances) because of noise in the dataset where the input PAR did not match the leaf surface PAR, and all errors were forced into estimation of $r_{bW}$. Hence, for further analysis of $r_{bW}$ and estimation of parameters of equation 5 (main text) all values of $r_{bW} > 250$ s m$^{-1}$ and all negative values were excluded. Further, at low PAR (< 200 µmol m$^{-2}$ s$^{-1}$) there was wide variation in $r_{bW}$ likely because with a low energy input any errors in the other microclimatic inputs and parameters will increase in importance. Hence, $r_{bW}$ estimated when PAR < 200 µmol m$^{-2}$ s$^{-1}$ were also excluded.

$r_{bW}$ was parameterized in two forms, with one or two varying parameters (equations S9 and S10)

$$r_{b,W} = 2/a\left({}^{U}/_{W}\right)^{0.5} \quad \text{(S9)}$$

$$r_{b,W} = 2/a\left({}^{U}/_{W}\right)^{b} \quad \text{(S10)}$$

Equation S10 showed a better fit to the data than equation S9 (Figure S3). Parameterization was performed separately for *Miconia* and *Alchornea*, and parameter *a* was similar for both species, with different values for *b* (Figure S2).
